# *Cinnamomum migao* Active Extracts Ameliorate Acute Myocardial Ischemia via Modulation of the HIF-1α/Nrf2/NF-κB/MAPK Pathway Downstream of Oxidative Stress

**DOI:** 10.3390/antiox15080970

**Published:** 2026-08-05

**Authors:** Xiaofen Li, Yinju Zhang, Wenxia Dai, Ming Xia, Lang Zhou

**Affiliations:** 1 School of Basic Medical Sciences, Guizhou University of Traditional Chinese Medicine, Guiyang 550025, China; 18785129704@163.com (X.L.); daiwen590@outlook.com (W.D.);; 2 State Key Laboratory of Discovery and Utilization of Functional Components in Traditional Chinese Medicine, Guiyang 550014, China; yinju.zhang@outlook.com; 3 Natural Products Research Center of Guizhou Province, Guizhou Medical University, Guiyang 550014, China

**Keywords:** *Cinnamomum migao* H. W. Li, acute myocardial ischemia, HIF-1α/Nrf2/NF-κB/MAPK, oxidative stress, inflammation

## Abstract

Acute myocardial ischemia (AMI) is a life-threatening cardiovascular disorder characterized by excessive oxidative stress and persistent inflammatory cascades, yet safe multi-target natural therapeutic agents remain scarce. *Cinnamomum migao* is a well-known Miao ethnic medicine used for cardiovascular conditions, yet its cardioprotective effects and mechanisms remain largely unclear. This study investigated the efficacy and underlying mechanism of *C. migao* ethyl acetate extract (MGE) against AMI. MGE significantly improved the viability of H9c2 cardiomyocytes subjected to OGD/R injured. UPLC-MS/MS and molecular networking identified 30 constituents in MGE, among which sesquiterpenoids predominated. In ISO-induced AMI rats, MGE dose-dependently mitigated myocardial injury, as reflected by reduced ST-segment elevation, serum CK-MB and LDH levels, and alleviated histopathological damage. MGE enhanced SOD and CAT activities, decreased MDA content, and inhibited the secretion of TNF-α, IL-6 and IL-1β. Mechanistically, MGE downregulated the expression of NOX4, HIF-1α, p38 MAPK and NF-κB p65, while activating the Nrf2/HO-1 pathway. Oxyphyllenone A and magnodelavin C were identified as key active sesquiterpenoids that stably bound to IL-17 and TNF. Collectively, MGE alleviates AMI injury via anti-oxidative, anti-hypoxic, and anti-inflammatory effects through modulation of the HIF-1α/Nrf2/NF-κB/MAPK axis downstream of oxidative stress, with sesquiterpenoids serving as its key bioactive components. This work provides robust experimental evidence supporting *C. migao* as a promising natural antioxidant candidate for the prevention and treatment of AMI.

## 1. Introduction

Cardiovascular diseases constitute a major global public health challenge, with ischemic heart disease being the leading cause of mortality worldwide [1]. Acute myocardial ischemia (AMI) originates from insufficient coronary perfusion and oxygen deprivation, which triggers irreversible myocardial injury and may further progress to angina pectoris, myocardial infarction, heart failure, and even sudden cardiac death [2,3]. Current clinical therapies for AMI mainly include revascularization strategies (percutaneous coronary intervention, coronary artery bypass grafting) and pharmacological interventions (thrombolysis, antiplatelet therapy, lipid-lowering therapy) [4,5]. Percutaneous coronary intervention represents the first-line treatment for acute myocardial infarction but is limited by low timely reperfusion rates and restricted eligibility in elderly and high-risk patients [5,6,7]. Thrombolytic therapy achieves only 50–70% vascular recanalization, accompanied by severe residual stenosis and a high bleeding risk [8]. Moreover, long-term administration of conventional drugs often induces adverse effects such as drug resistance and hepatorenal toxicity, while high medical costs compromise patient compliance [9]. Thus, developing safe, effective, affordable, and multi-target therapeutic agents is urgently needed for the prevention and treatment of AMI.

The pathological mechanism of acute myocardial ischemia is complex and involves oxidative stress, inflammatory responses, apoptosis, mitochondrial dysfunction, and calcium overload [10,11,12,13,14]. Among these, oxidative stress, inflammatory responses, and cardiomyocyte apoptosis represent the three core and interrelated mechanisms, which form a vicious cycle and collectively mediate progressive myocardial injury and cardiac dysfunction. Myocardial ischemia rapidly induces mitochondrial dysfunction and massive accumulation of reactive oxygen species (ROS) [15]. Excessive ROS trigger lipid peroxidation, protein oxidation, and DNA damage, destroy cellular structures, impair the vascular endothelium, and further aggravate myocardial ischemia [16]. Meanwhile, the injured myocardium releases damage-associated molecular patterns (DAMPs), which activate TLRs and the NLRP3 inflammasome, promote the release of pro-inflammatory cytokines such as IL-1β, TNF-α, and IL-6, mediate immune cell infiltration, and exacerbate inflammatory necrosis [17]. Sustained oxidative stress and inflammatory signals can initiate the mitochondrial apoptotic pathway by regulating Bcl-2 family proteins and releasing cytochrome c, as well as the death receptor pathway, thereby triggering the caspase cascade and inducing extensive cardiomyocyte apoptosis [17]. This directly results in reduced cardiomyocyte numbers and disrupted myocardial contractile units, and ultimately contributes to severe complications such as heart failure and arrhythmia. These three processes regulate and interact with each other synergistically, constituting the core pathological network of acute myocardial ischemic injury and providing an important theoretical basis for multi-target drug intervention.

Migao is the mature fruit of *Cinnamomum migao* (Lauraceae). As an endemic species in China, it is renowned as a famous *Daodi* herb in Guizhou Province and is recognized as one of the “Top Ten Miao Medicines”. As a classic Miao medicine for regulating *qi* and alleviating pain, it has a long history of traditional application in regions inhabited by the Miao, Bouyei, and Zhuang ethnic groups, and has been officially documented in both the Chinese Materia Medica (Miao Medicine Volume) and the *Quality Standards for Chinese and Ethnomedicines of Guizhou Province* [18,19]. Traditionally, *C. migao* possesses the efficacies of warming the middle *jiao*, dispersing cold, regulating *qi*, and relieving pain. It is primarily utilized in the prevention and treatment of acute myocardial ischemia (AMI) and its associated symptoms caused by *qi* stagnation and blood stasis, such as chest *bi* (chest obstruction), chest oppression, pricking pain, and palpitations. Furthermore, it acts as an essential raw material for multiple marketed Miao patent medicines, such as Liqi Huoxue Dropping Pills, Xinnaoning Capsules, Xinwei Zhitong Capsules, and Jinhoujian Spray.

Modern phytochemical investigations have revealed that the principal chemical constituents of *C. migao* are essential oils and sesquiterpenoids [19,20,21,22,23,24]. However, its cardioprotective effects against acute myocardial ischemia and the underlying molecular mechanisms have not yet been systematically elucidated. Therefore, this study aims to investigate the cardioprotective potential of *C. migao* and further explore its mechanisms targeting oxidative stress, inflammatory responses, and apoptotic pathways. Our findings will provide a scientific basis for developing novel natural therapeutic agents against myocardial ischemia.

## 2. Materials and Methods

### 2.1. Reagents

Isoprenaline HCl (ISO: ≥99.9%) was purchased from Shanghai Aladdin Biochemical Technology Co., Ltd. (Shanghai, China). CK-MB, TNF-α, IL-6 and IL-1β Elisa kits, as well as LDH, MDA, SOD and CAT biochemical kits, were all purchased from Wuhan Elabscience Biotechnology Co., Ltd. (Wuhan, China). Antibodies against HIF-1α, HO-1, NK-κB p65 and NOX4 were all purchased from Chengdu Zhengneng Biotechnology Co., Ltd. (Chengdu, China). Nrf2 and p38 MAPK antibodies were purchased from Affinity Biosciences Company (Cincinnati, OH, USA). Beta-actin antibody was purchased from Wuhan Sanying Biotechnology Co., Ltd. (Wuhan, China). Fetal bovine serum (FBS) and Dulbecco’s modified Eagle’s medium (DMEM) were purchased from Wuhan Pricella Biotechnology Co., Ltd. (Wuhan, China).

### 2.2. Preparation of C. migao Ethyl Acetate Extract (MGE)

The mature fruits of *C. migao* were collected from Luodian County, Guizhou Province, China. The plant material was authenticated by Dr. Jing-Zhong Chen (Guizhou University of Traditional Chinese Medicine). A voucher specimen (GZLD20251011) has been deposited at the Natural Products Research Center of Guizhou Province.

The air-dried and pulverized plant material (11.0 kg) was extracted with methanol (MeOH) under reflux three times (2 h each). The solvent was removed under reduced pressure to afford a crude extract. This extract was then suspended in H_2_O and successively partitioned with petroleum ether and ethyl acetate (EtOAc). The respective organic layers were concentrated under reduced pressure and dried in vacuo to yield the petroleum ether (MGP) and ethyl acetate fractions (MGE), which were subsequently stored at −20 °C until further use.

### 2.3. LC-MS/MS Analysis

Liquid chromatography-tandem mass spectrometry (LC-MS/MS) analysis of the *C. migao* subfractions was performed on a Dionex Ultimate 3000 RSLC system (Thermo Fisher Scientific, Waltham, MA, USA) coupled with a heated electrospray ionization (HESI-II) dual source. Chromatographic separation was achieved utilizing a Waters Acquity UPLC HSS T3 column (100 × 2.1 mm, 1.8 μm) (Waters Corporation, Milford, MA, USA). The mobile phase consisted of 0.1% aqueous formic acid (*v*/*v*, solvent A) and acetonitrile containing 0.1% formic acid (solvent B). Elution was conducted at a constant flow rate of 0.3 mL/min with the following gradient profile: initial hold at 5% B for 2 min, linear increase to 95% B from 2 to 42 min, an isocratic phase at 95% B until 47 min, and a final re-equilibration step at 5% B (47.1–50 min).

### 2.4. Molecular Networking

Mass spectra were recorded over an *m*/*z* range of 100–1500. Following data acquisition, raw files were transformed into the mzXML format via RawConverter. To construct the molecular network, the converted data were uploaded to the Global Natural Products Social Molecular Networking (GNPS) web infrastructure (http://gnps.ucsd.edu, accessed on 11 March 2023). Network generation parameters were configured with a precursor ion mass tolerance of 2.0 Da and a fragment ion tolerance of 0.5 Da. Edges were filtered to retain only those exhibiting a cosine score exceeding 0.7 and a minimum of six matched fragment ions. Cytoscape (version 3.10) was utilized for the final visualization of the spectral networks. The complete GNPS job and corresponding results are publicly accessible at http://gnps.ucsd.edu/ProteoSAFe/status.jsp?task=6e4597b967ed4963b8d351ab09ce8c80, accessed on 11 March 2023.

### 2.5. Cell Culture and Oxygen–Glucose Deprivation/Reoxygenation (OGD/R) Assay

The H9c2 rat cardiomyocytes were acquired from the Shanghai iCell Bioscience (Shanghai, China) and cultured in DMEM supplemented with 10% fetal bovine serum (FBS) and 1% penicillin-streptomycin. The cells were incubated at 37 °C in an environment containing 5% carbon dioxide. Upon reaching 80–90% confluence, H9c2 cells in the exponential growth phase were seeded into 96-well plates at a density of 5 × 10^3^ cells/well and incubated for 24 h. The cells were then treated with the test compounds (50 μM) and co-incubated for an additional 24 h. To induce oxygen–glucose deprivation, the original medium was discarded and replaced with glucose-free DMEM. The cells were transferred to a tri-gas incubator and subjected to hypoxia (1% O_2_, 5% CO_2_, and 94% N_2_) for 4 h. Following hypoxic exposure, the medium was replaced with complete high-glucose DMEM supplemented with 10% fetal bovine serum (FBS). The cells were subsequently returned to a normoxic incubator (37 °C, 95% air, and 5% CO_2_) for 2 h of reoxygenation. Cell viability was evaluated using a Cell Counting Kit-8 (CCK-8) assay. The absorbance (OD value) of each well was measured at 450 nm using a microplate reader to calculate the cell survival rate.

### 2.6. Animals and Treatment

Male Sprague–Dawley rats (7–8 weeks old, 200–220 g) were obtained from Beijing SPF Biotechnology Co., Ltd. (Beijing, China). The animals were housed at a constant temperature (20 ± 2 °C) with a 12-h light-dark cycle. After 7 days of adaptive feeding, 48 rats were randomly divided into 6 groups: blank control group (normal saline); model group (normal saline + ISO); positive propranolol group (propranolol 10 mg/kg + ISO); MGE groups (MGE 200 mg/kg + ISO; and MGE 100 mg/kg + ISO; MGE 50 mg/kg + ISO). All drug groups were intragastrically administered for a total of 9 days. ISO is a non-selective β-adrenergic agonist. High-dose ISO excessively activates cardiac β1 receptors, causing tachycardia, hypercontractility and sharply increased myocardial oxygen consumption, thereby triggering an oxygen supply–demand imbalance and acute myocardial ischemia. Hyperactivated β-signaling further drives ROS overproduction, lipid peroxidation, inflammatory factor release and mitochondrial dysfunction, resulting in myocardial edema, inflammation and necrosis that mimic clinical AMI pathology. The blank control group and the model group were injected with the same volume of normal saline. Except for the blank group, all other groups were subcutaneously injected with ISO (85 mg/kg) into the abdomen 1 h after intragastric administration on the 8th and 9th days to establish a myocardial ischemia model. All animal studies were conducted under the authorization of the Animal Ethics Committee of Guizhou University of Traditional Chinese Medicine (Approval No: TCM-2025-020).

### 2.7. Assessment of the Electrocardiogram (ECG) and Cardiac Enzymes

On the 9th day of the experiment, 1 h after the rats were intraperitoneally injected with ISO, they were anesthetized with urethane and placed in a supine position, with needle electrodes implanted subcutaneously in their limbs. Subsequently, the electrodes were connected to the BL4-20S biological signal acquisition and analysis system for measuring lead II electrocardiogram (ECG) and monitoring changes in the ΔST segment of the rats. Then, abdominal aortic blood was collected from the rats. After the blood was allowed to stand at room temperature for 1 h, it was centrifuged at 6000× *g* for 10 min at 4 °C. Following serum separation, the levels of CK-MB and LDH were detected using the corresponding kits.

### 2.8. Assessment of Inflammation and Oxidative Stress Markers

Serum was collected from rats in each group. The levels of serum inflammatory factors (TNF-α, IL-6, IL-1β) were detected using Elisa kits, and the levels of serum oxidative stress marker (free and protein-bound MDA) and antioxidant enzymes (SOD, CAT) were detected using biochemical kits by following the manufacturer’s instructions of Wuhan Elabscience Biotechnology Co., Ltd.

### 2.9. Histopathological Examination

Rat heart tissues were harvested, rinsed with normal saline and blotted dry with filter paper. The tissue was fixed with 4% paraformaldehyde, then dehydrated and embedded, followed by continuous sectioning at 4 μm. After hematoxylin-eosin (H&E) staining, dehydration and mounting were performed, and myocardial pathological changes were observed under a microscope. Myocardial pathological lesions were blindly assessed under light microscopy using a modified 0–5 semi-quantitative scoring system, with independent evaluation of cardiomyocyte edema, interstitial inflammatory infiltration and cardiomyocyte necrosis:

0: No pathological changes; no cardiomyocyte edema, no inflammatory infiltration, intact myocardial fibers.

1: Mild cardiomyocyte edema, rare inflammatory cells, no cardiomyocyte necrosis.

2: Mild-to-moderate cardiomyocyte edema, sparse interstitial inflammatory infiltration, no obvious necrosis.

3: Moderate cardiomyocyte edema, scattered inflammatory cell infiltration, focal cardiomyocyte degeneration.

4: Severe cardiomyocyte edema, extensive inflammatory infiltration, multifocal cardiomyocyte necrosis.

5: Diffuse severe cardiomyocyte edema, massive inflammatory cell infiltration, widespread myofibril dissolution and confluent myocardial necrosis.

### 2.10. Network Pharmacology Analysis

The chemical constituents characterized via LC-MS/MS and molecular networking were submitted to the SwissTargetPrediction database (www.swisstargetprediction.ch, accessed on 13 April 2026), yielding 387 putative gene targets. Simultaneously, 557 disease-related targets for “acute myocardial ischemia” were retrieved from GeneCards (https://www.genecards.org/, accessed on 13 April 2026). Overlapping targets were acquired via the Venny 2.1 web tool (https://bioinfogp.cnb.csic.es/tools/venny/index.html, accessed on 13 April 2026). For protein–protein interaction (PPI) profiling, these consensus targets were analyzed utilizing the STRING database (https://string-db.org, accessed on 13 April 2026), followed by network construction and visualization in Cytoscape (version 3.10.3). To further explore the underlying biological mechanisms, GO functional and KEGG pathway enrichment analyses were executed using the DAVID database (https://davidbioinformatics.nih.gov/, accessed on 14 April 2026). The corresponding graphical representations were plotted via a bioinformatics web server (https://www.bioinformatics.com.cn/, accessed on 14 April 2026).

### 2.11. Western Blot Assay

Total proteins were extracted from rat heart tissues to detect the expression levels of target proteins (HIF-1α, HO-1, NK-κB p65, NOX4, Nrf2, and p38 MAPK) under different treatment conditions. Briefly, heart tissues were fully homogenized on ice in RIPA lysis buffer containing 1× protease inhibitor cocktail and phosphatase inhibitor cocktail. After sufficient lysis, the homogenate was centrifuged at 12,000× *g* for 15 min at 4 °C, and the supernatant was collected as total protein extracts. After quantification by BCA method, protein samples were separated by sodium dodecyl sulfate-polyacrylamide gel electrophoresis (SDS-PAGE) and polyvinylidene difluoride membrane (PVDF). After blocking with 5% bovine serum albumin, the membranes were incubated with specific primary antibodies at the corresponding concentrations overnight at 4 °C, followed by incubation with secondary antibodies. The results were determined by an enhanced chemiluminescence (ECL) system and quantitatively analyzed using ImageJ 1.51j8 software.

### 2.12. Molecular Docking Analysis of the Active Constituents

Molecular docking simulations were conducted using AutoDock 4 software to investigate the binding modes of compounds **3** and **12** with key inflammatory targets. The three-dimensional (3D) crystal structures of the target proteins, IL-17 (PDB ID: 3VBC) and TNF (PDB ID: 5MU8), were retrieved from the RCSB Protein Data Bank (https://www.rcsb.org/). Concurrently, the 3D geometries of compounds **3** and **12** were generated utilizing Chem3D 22.0 software. Following the docking procedures, the conformations with the optimal docking scores were visualized and analyzed using PyMOL 2.4. Furthermore, two-dimensional (2D) diagrams illustrating the protein–ligand interactions were generated via the LigPlot^+^ Molecular Graphics System (version 2.3.1).

### 2.13. Statistical Analysis

Experimental data were analyzed using SPSS 20.0 software. The results were expressed as mean ± standard deviation. For data that conformed to normal distribution, one-way analysis of variance (ANOVA) was used, followed by Dunnett’s test and Tukey’s test. For data that did not conform to normal distribution, independent-samples *t*-test was used. The criterion for determining statistical significance was *p* < 0.05 or *p* < 0.01.

## 3. Results

### 3.1. In Vitro Bioactivity Screening

**Figure 1:**
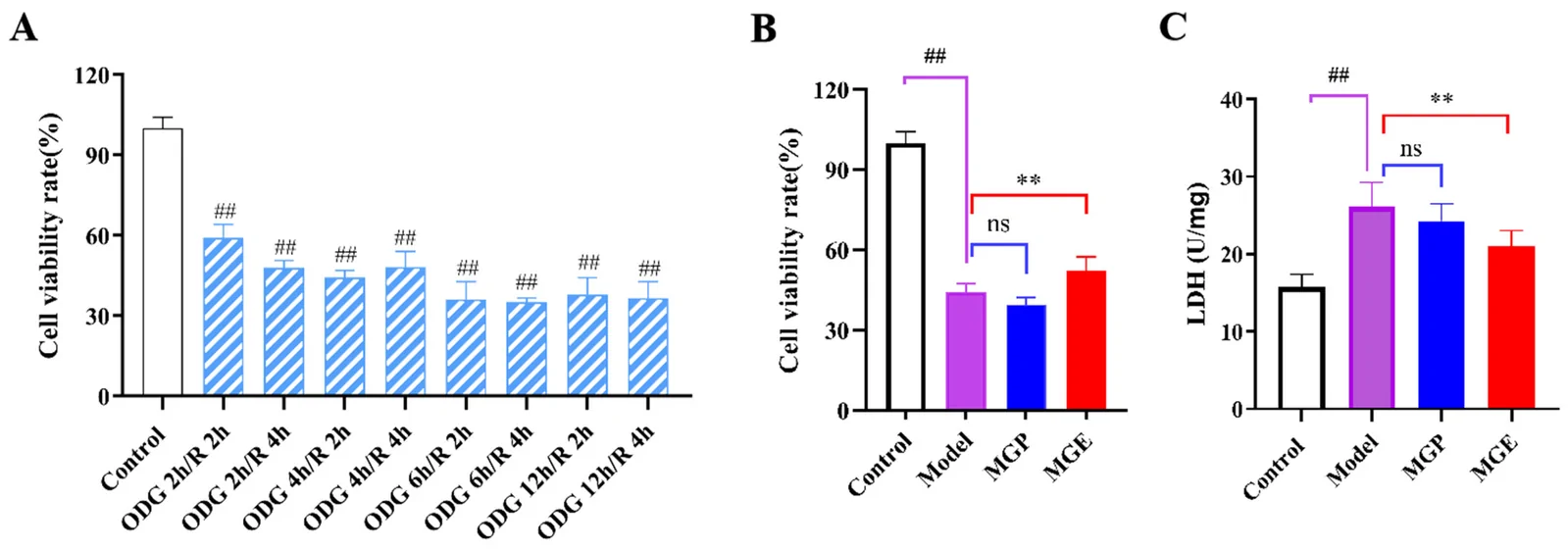
Optimization of OGD/R modeling and efficacy evaluation of *C. migao*. (**A**) Optimization of oxygen–glucose deprivation/reoxygenation (OGD/R) duration (*n* = 6). (**B**,**C**) Cell viability and LDH level of H9c2 cardiomyocytes subjected to OGD 4 h/R 2 h following MGP and MGE treatment (*n* = 6). ^##^ *p* < 0.01 vs. control; ** *p* < 0.01 vs. model.

To establish an optimal OGD/R-induced H9c2 cardiomyocyte injury model, a systematic screening of OGD/R temporal parameters was initially performed. Various combinations of OGD durations (2, 4, 6, 12, and 24 h) coupled with reoxygenation periods (2 and 4 h) were rigorously evaluated (Figure 1A). Based on the cellular injury outcomes, the OGD 4 h/R 2 h protocol was ultimately selected as the standard condition for evaluating the in vitro anti-AMI efficacy of the petroleum ether (MGP) and ethyl acetate (MGE) fractions extracted from Migao. Compared with the untreated model group (44.28%), treatment with MGP (39.50%) and MGE (54.65%) at a concentration of 200 μg/mL showed that MGE exerted significant cytoprotective effects (Figure 1B). Consistent with the cell viability results, LDH release assay further verified the protective effect of MGE. As illustrated in Figure 1C, LDH activity was markedly elevated in the model group relative to the control group, whereas MGE treatment significantly reduced LDH leakage compared with the model group. These findings confirm that the MGE fraction represents the primary pharmacologically active fraction.

### 3.2. Characterization of Compounds in MGE

**Figure 2:**
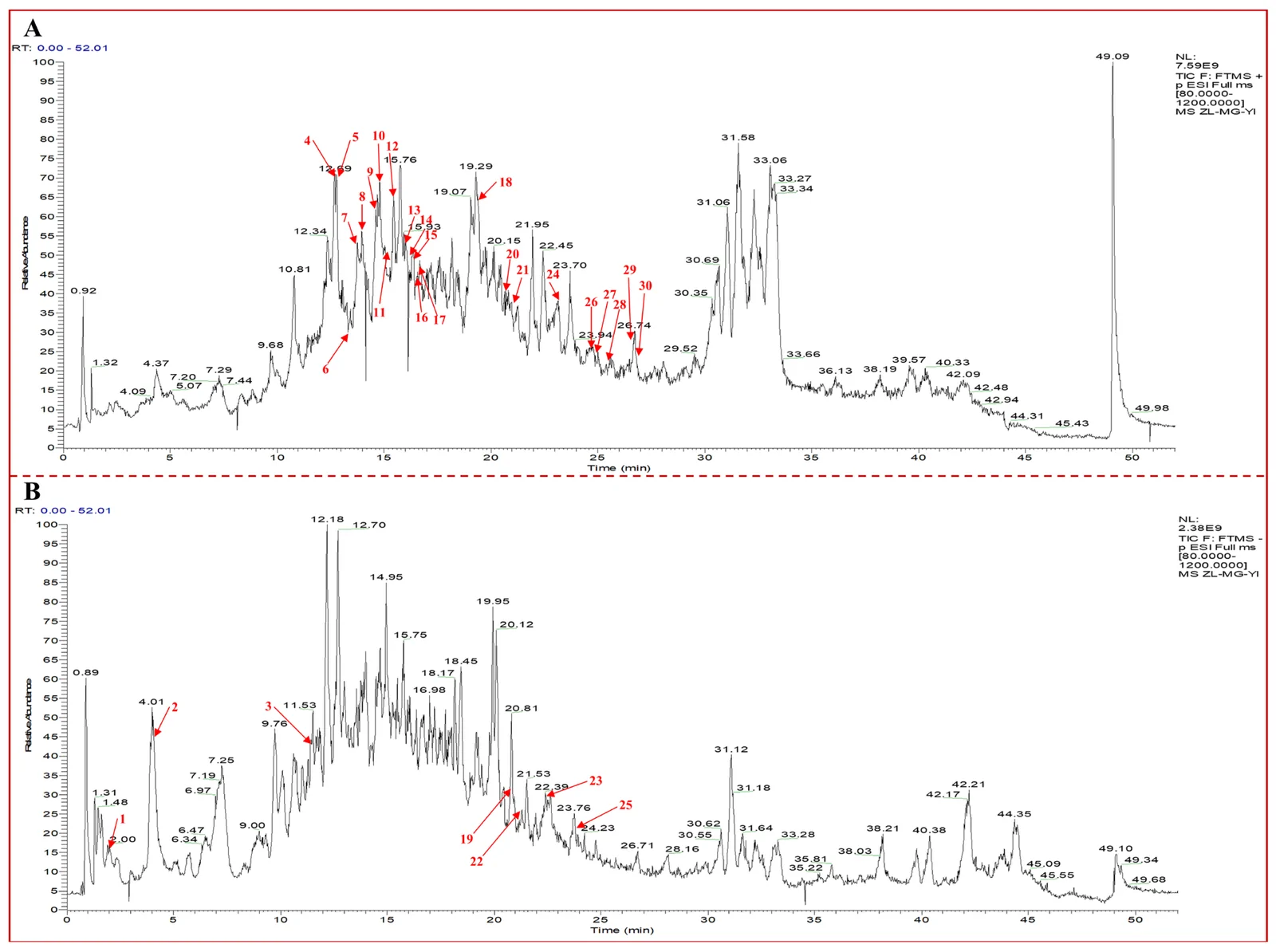
UPLC–MS/MS total ion chromatogram (TIC) of the ethyl acetate fraction from *C. migao*. (**A**) Positive ion mode; (**B**) negative ion mode.

**Figure 3:**
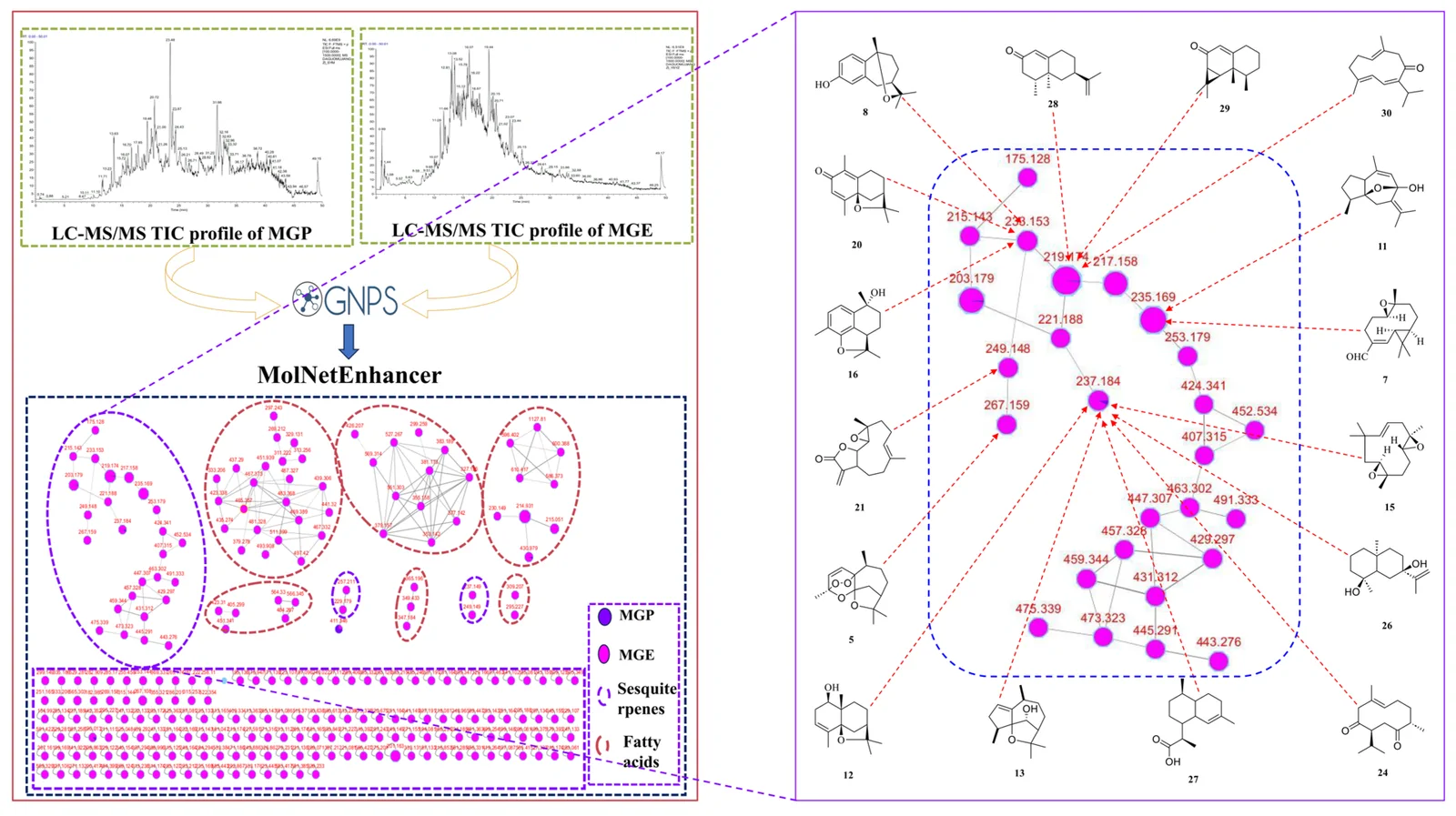
Molecular networking of MGE.

Following data preprocessing, the tandem mass spectrometry (MS/MS) spectra (Figure 2) were uploaded to the Global Natural Products Social Molecular Networking (GNPS) platform to construct a molecular network for MGE (Figure 3), which was subsequently visualized utilizing Cytoscape software. In this network, compounds with homologous chemical scaffolds and similar fragmentation patterns were clustered into distinct molecular families, thereby greatly facilitating structural annotation and dereplication. By systematically matching precursor ion *m*/*z* values and MS/MS fragmentation patterns with reference standards, literature data, and an in-house mass spectral library, a total of 30 constituents were unambiguously identified in MGE, including six monoterpenes and 24 sesquiterpenoids (Supplementary Figure S1 and Table S1).

### 3.3. MGE Protects the Heart from Ischemic Injury

**Figure 4:**
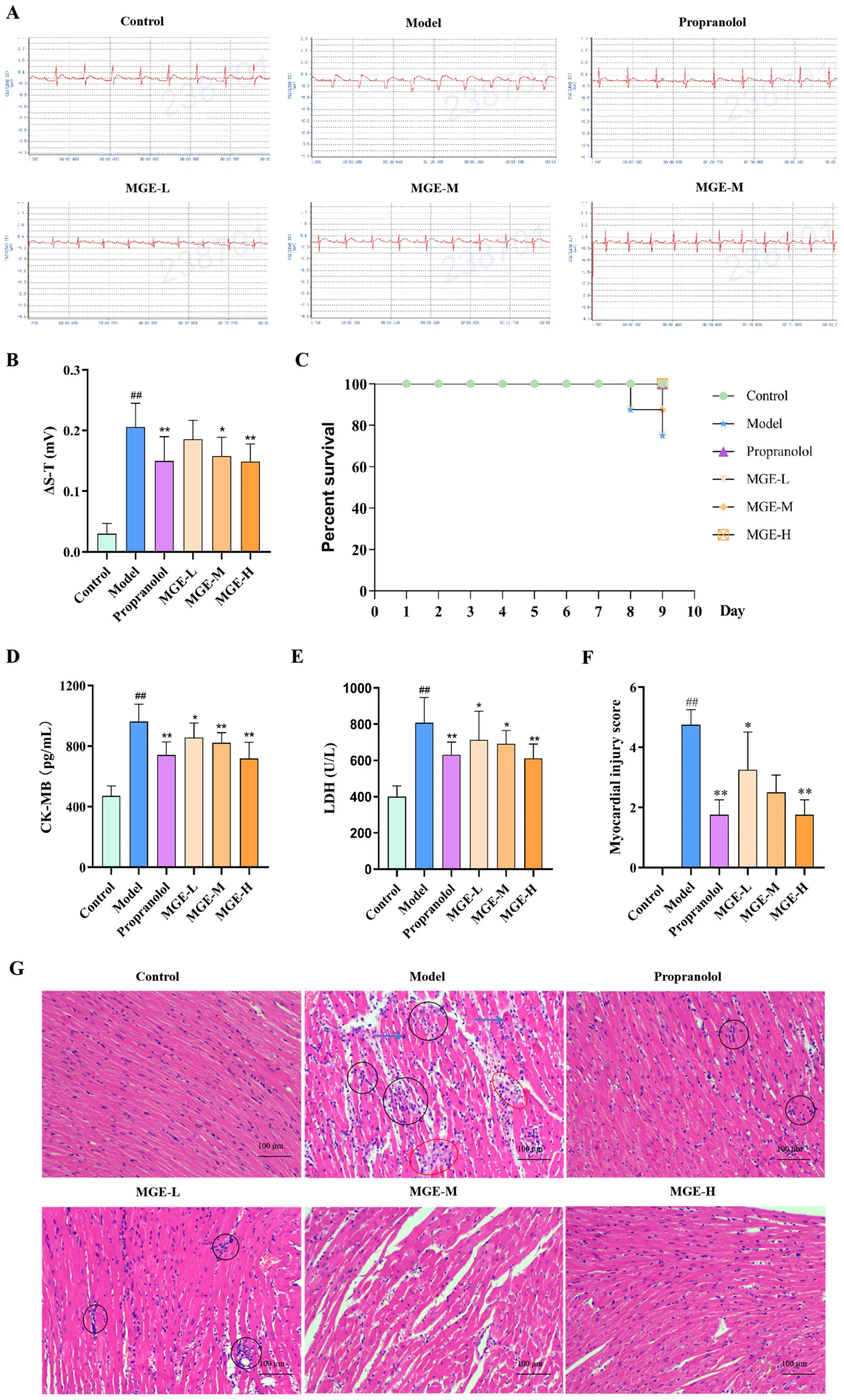
MGE protects the heart from ischemic injury in rats. (**A**) Representative ECG traces. (**B**) ΔS−T change values of ECG (*n* = 6). (**C**) Survival curves of rats (*n* = 8). (**D**,**E**) Serum levels of CK−MB and LDH (*n* = 6). (**F**,**G**) Myocardial injury score and histopathological morphology of myocardial tissues observed by HE staining (*n* = 4) (scale bar = 50 μm). ^##^ *p* < 0.01 vs. control; * *p* < 0.05, ** *p* < 0.01 vs. model.

Electrocardiographic monitoring (Figure 4A,B) showed regular ECG morphology and no obvious ST-segment deviation in the control group. In contrast, the model group exhibited marked ST-segment elevation, confirming the successful establishment of the myocardial ischemia model. Relative to the model group, the extent of ST-segment elevation was significantly alleviated in the propranolol group and all MGE-treated groups in a dose-dependent manner. Notably, the high-dose MGE group produced a therapeutic effect comparable to that of the positive control. Survival observation within 10 consecutive days revealed that the model group displayed decreased survival rate, whereas MGE treatment improved the survival status of ischemic rats (Figure 4C). Serum enzymatic detection revealed that the levels of CK-MB and LDH, two established biomarkers for myocardial injury, were remarkably increased in the model group compared with the control group. Administration of propranolol and all doses of MGE significantly reduced the levels of these two enzymes, with the most prominent improvement detected in the MGE-H group (Figure 4D,E). Consistent with serum biochemical results, semi-quantitative histological scoring further validated the cardioprotective effect of MGE. The model group presented a markedly elevated myocardial injury score, while propranolol and gradient concentrations of MGE notably lowered the injury score (Figure 4F). Histological examination by HE staining showed intact myocardial architecture and neatly arranged myocardial fibers in the control group. In the model group, myocardial fibers were disorganized, accompanied by overt cardiomyocyte edema, enlarged intercellular spaces, partial nuclear loss, interstitial inflammatory cell infiltration, and obvious necrotic cardiomyocytes. Compared with the model group, MGE treatment effectively restored the orderly arrangement of myocardial fibers and attenuated cardiomyocyte edema and inflammatory infiltration (Figure 4G).

### 3.4. MGE Attenuates Inflammation and Oxidative Stress Against Ischemic Injury

**Figure 5:**
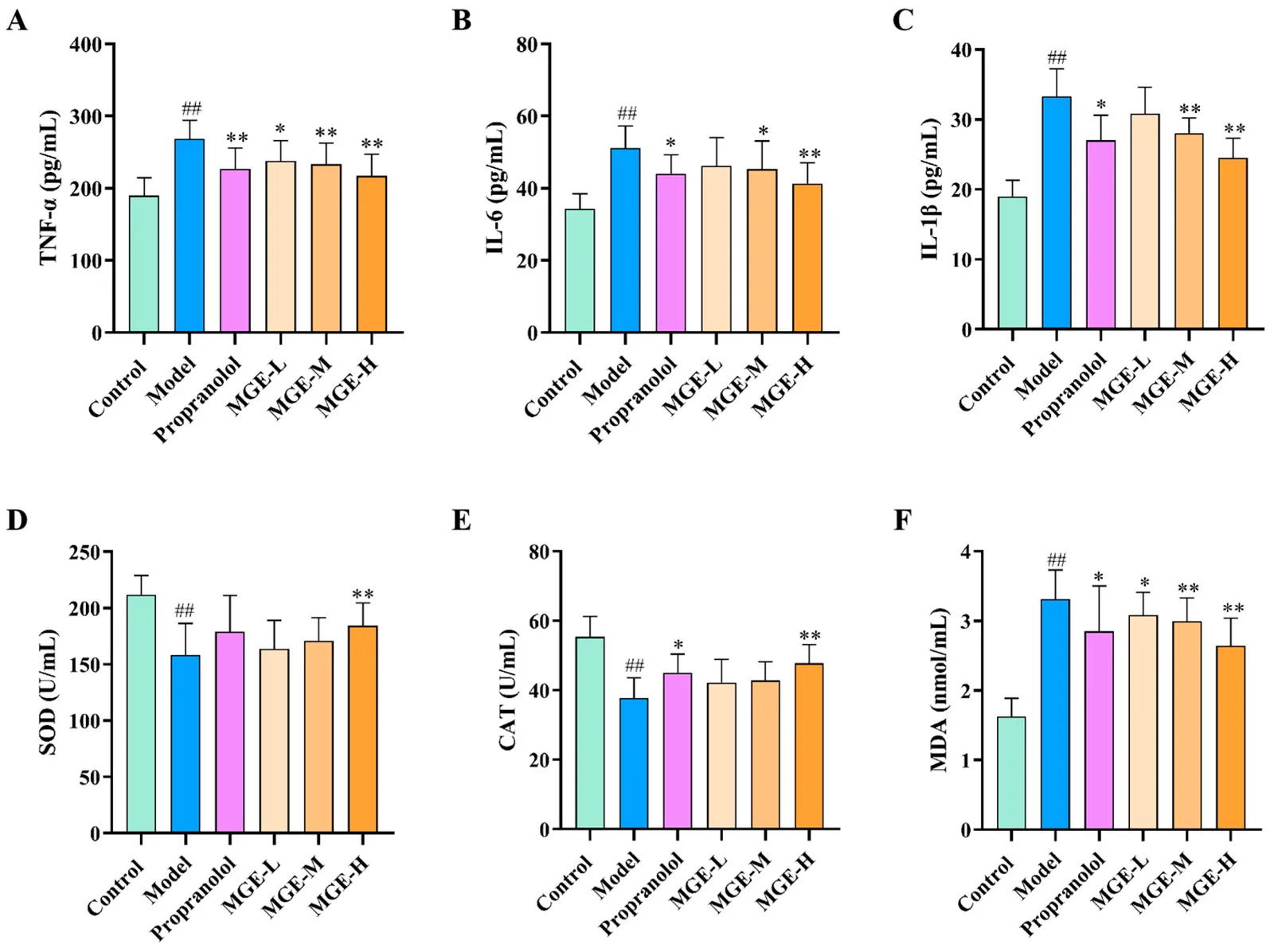
MGE ameliorates inflammation and oxidative stress in rats with ischemic injury. (**A**–**C**) Serum levels of TNF-α, IL-6 and IL-1β (*n* = 6). (**D**–**F**) Serum levels of SOD, CAT and MDA (*n* = 6). ^##^ *p* < 0.01 vs. control; * *p* < 0.05, ** *p* < 0.01 vs. model.

As illustrated in Figure 5A–C, serum levels of the pro-inflammatory cytokines TNF-α, IL-6, and IL-1β were markedly elevated in the model group relative to the control group, indicating severe systemic inflammation associated with myocardial ischemic injury. Compared with the model group, treatment with propranolol and all doses of MGE significantly reduced TNF-α, IL-6, and IL-1β levels in a dose-dependent manner, with the most striking reduction observed in the MGE-H group. Results of oxidative stress assessment (Figure 5D–F) revealed that serum activities of the antioxidant enzymes SOD and CAT were significantly decreased, whereas the level of the oxidative product MDA was distinctly increased in the model group versus the control group, demonstrating severe oxidative stress imbalance in model rats. Relative to the model group, propranolol and all MGE treatments significantly enhanced SOD and CAT activities and reduced MDA levels. The MGE-H group exhibited the most optimal effect in restoring oxidative stress parameters, confirming a marked anti-oxidative injury activity. Collectively, these findings demonstrate that MGE protects against myocardial ischemic injury by suppressing the release of pro-inflammatory cytokines to alleviate inflammatory responses, and by enhancing antioxidant enzyme activities to restore oxidative stress balance.

### 3.5. Prediction of Key Target Proteins of MGE Against AMI

**Figure 6:**
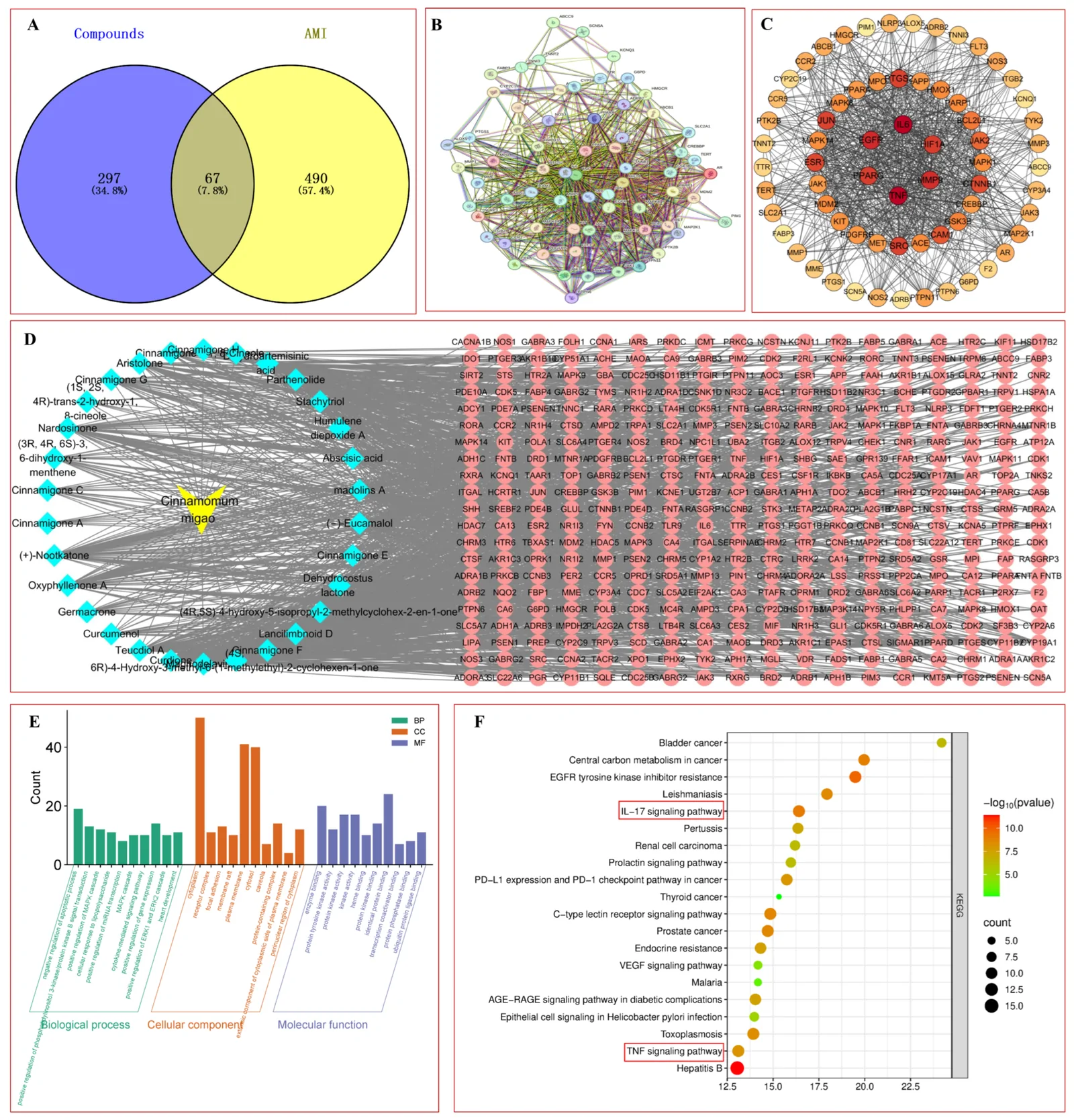
Network pharmacology analysis of active components from *C. migao* against AMI. (**A**) Venn diagram of common targets between *C. migao* active components and AMI. (**B**) PPI network of *C. migao* active components and AMI targets. (**C**) Core target map. (**D**) *C. migao*-components-targets interaction network. (**E**) GO functional enrichment analysis of potential therapeutic targets. (**F**) KEGG pathway enrichment analysis of potential targets.

A network pharmacology approach was employed to elucidate the potential targets of the 30 characterized constituents in MGE. Systematic screening of the SwissTargetPrediction database yielded 387 candidate targets. Concurrently, 557 disease-associated targets related to acute myocardial ischemia (AMI) were retrieved from the GeneCards database. Subsequent intersection analysis using a Venn diagram identified 67 overlapping genes between the constituent targets and AMI-related targets (Figure 6A–C). To intuitively visualize these complex pharmacological relationships, a “compound-target-pathway” network was constructed (Figure 6D). Furthermore, GO enrichment and KEGG pathway enrichment analysis revealed the top 20 significantly enriched pathways potentially modulated by the MGE constituents (Figure 6E,F). Notably, these included the IL-17 and TNF signaling pathways, both of which are closely implicated in the pathogenesis of AMI. Collectively, these network pharmacology predictions provide valuable mechanistic insights and a directional framework for future investigations into the anti-AMI efficacy of *C. migao*.

### 3.6. MGE Alleviates AMI Injury by Modulating the HIF-1α/Nrf2/NF-κB/MAPK Signaling Pathway Downstream of Oxidative Stress

**Figure 7:**
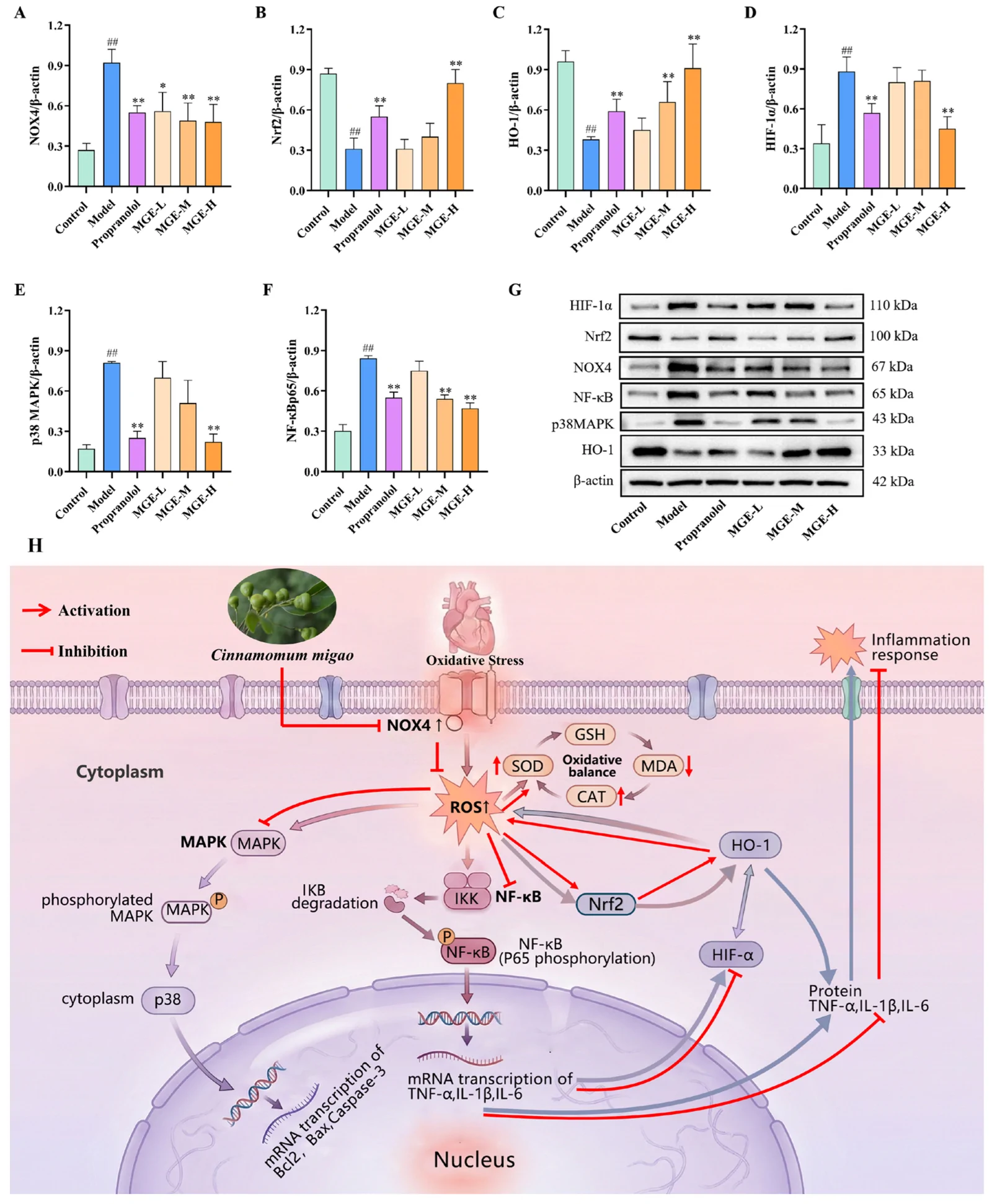
Effects of MGE on the expression of HIF-1α/Nrf2/NF-κB/p38 MAPK pathway-related proteins in rats with myocardial ischemia. (**A**–**F**) Quantitative analysis of HIF-1α, Nrf2, NF-κBp65, NOX4, p38 MAPK, and HO-1 protein expression in myocardial tissues (*n* = 5). (**G**) Representative Western blot bands of related proteins in myocardial tissues. ^##^ *p* < 0.01 vs. control; * *p* < 0.05, ** *p* < 0.01 vs. model. (**H**) Proposed molecular mechanisms of MGE in ameliorating AMI.

To further validate the signaling proteins predicted by network pharmacology and delineate the underlying molecular mechanism, we examined the expression of key regulators in myocardial tissue. As illustrated in Figure 7A–G, compared with the control group, the model group exhibited markedly elevated protein levels of the pro-oxidative enzyme NOX4, hypoxia-inducible factor HIF-1α, and core inflammatory mediators NF-κB p65 and p38 MAPK, accompanied by significantly reduced expression of the pivotal antioxidant transcription factor Nrf2 and its downstream effector HO-1. These results verified the marked activation of hypoxia, inflammation, and oxidative stress-associated signaling pathways, as well as the impairment of the endogenous antioxidant defense system in the myocardial ischemia model. Relative to the model group, propranolol and all doses of MGE significantly downregulated the protein expression of NOX4, HIF-1α, NF-κB p65 and p38 MAPK, while markedly upregulating Nrf2 and HO-1 expression in a clear dose-dependent manner. Collectively, these findings demonstrate that MGE confers multifaceted cardioprotective effects against myocardial ischemic injury, at least in part via modulating the HIF-1α/Nrf2/NF-κB/MAPK signaling axis to antagonize hypoxia, inflammation and oxidative stress.

### 3.7. In Vitro Evaluation of the Anti-AMI Activity of Isolated Sesquiterpenoids

**Figure 8:**
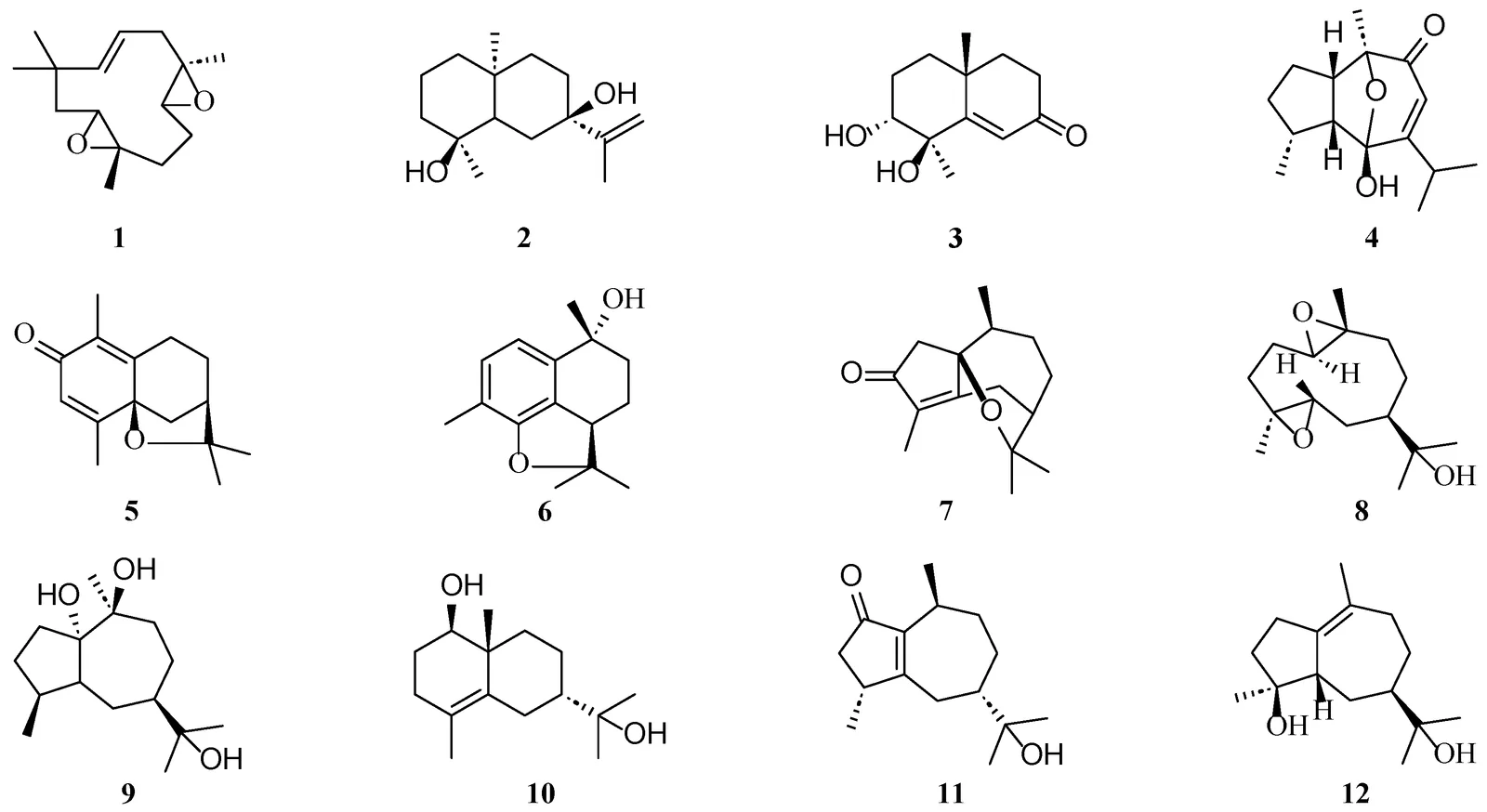
Chemical structures of the compounds in vitro anti-myocardial ischemic activity.

Subsequent phytochemical investigation of the MGE fraction led to the isolation and structural elucidation of twelve sesquiterpenoids, which were identified as humulene diepoxide A (**1**), teucdiol A (**2**), oxyphyllenone A (**3**), bulnesol (**4**), cinnamigone H (**5**), cinnamigone F (**6**), cinnamigone C (**7**), cinnamigone D (**8**), stachytriol (**9**), 7-epi-4-eudesmene-1β,11-diol (**10**), lancilimbnoid D (**11**), and magnodelavin C (**12**) (Figure 8). The NMR data are provided in the Supporting Information Material.

**Figure 9:**
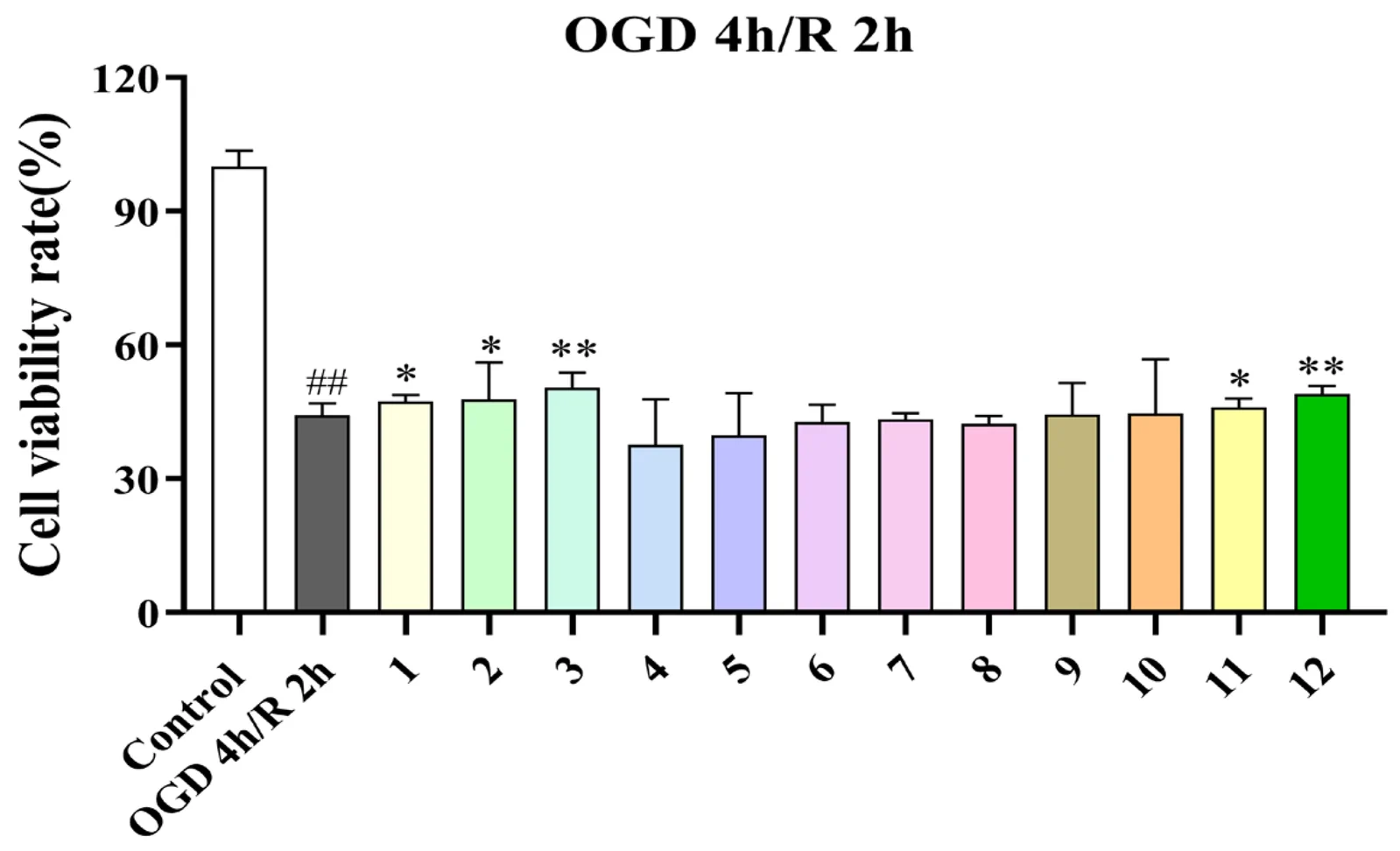
Effects of the isolated sesquiterpenoids (compounds **1**–**12**) on cell viability in H9c2 cell subjected to OGD 4 h/R 2 h. Cell viability was determined by CCK-8 assay (*n* = 6). ^##^ *p* < 0.01 vs. control; * *p* < 0.05, ** *p* < 0.01 vs. model.

Furthermore, the in vitro cardioprotective effects of the 12 isolated sesquiterpenoids on cell viability were assessed in H9c2 cardiomyocytes subjected to OGD 4 h/R 2 h. Compared with the control group, the OGD/R model group exhibited a significant reduction in cell viability. Compounds **1**, **2**, **3**, **11**, and **12** significantly increased cell viability under this condition, with compounds **3** and **12** showing the most pronounced protective effects. The remaining compounds (**4**–**10**) showed no significant improvement in cell viability. The comprehensive anti-ischemic activities of all evaluated compounds (**1**–**12**) are illustrated in Figure 9.

### 3.8. Molecular Docking Study

**Figure 10:**
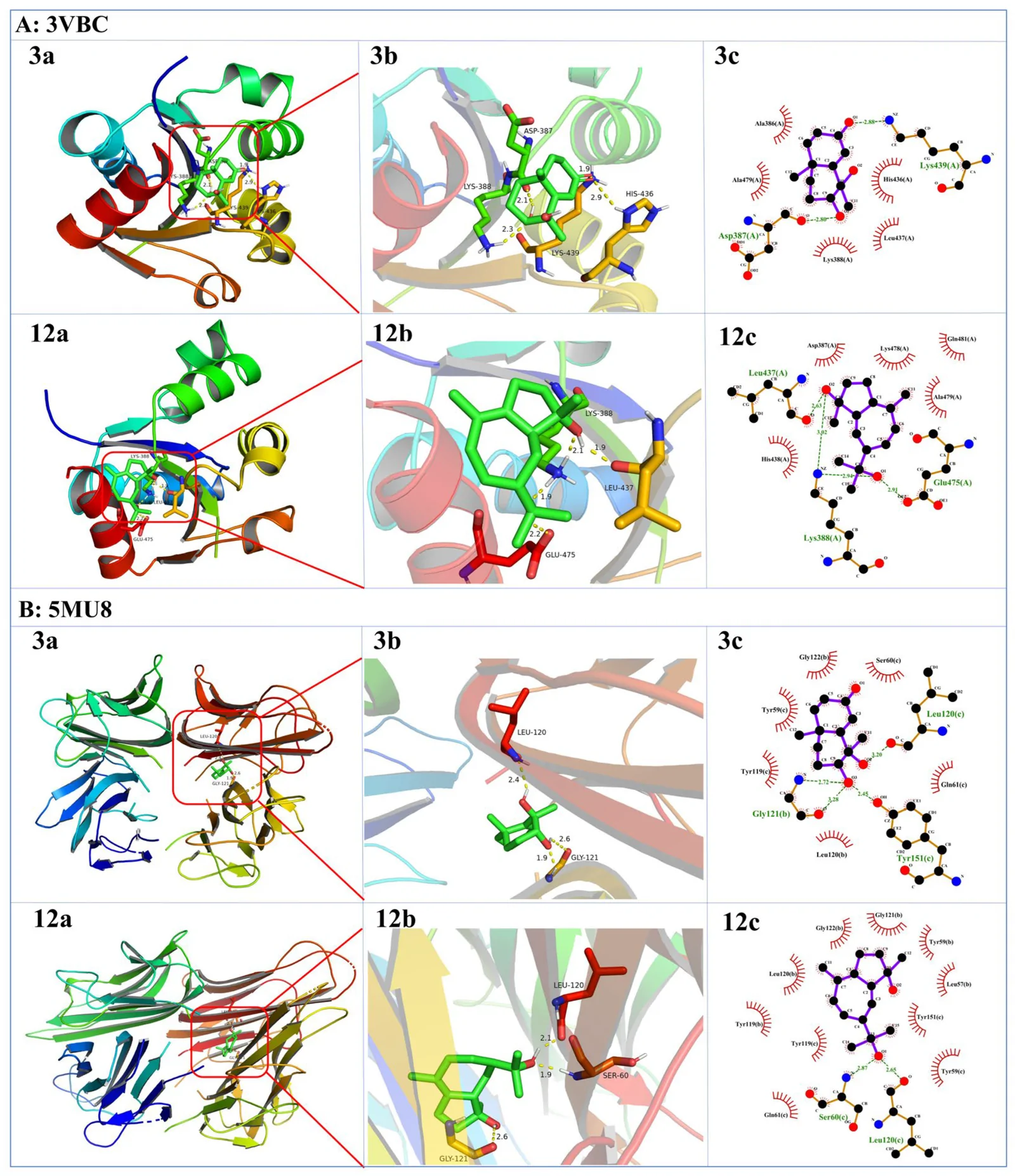
Molecular docking simulations illustrating the interactions between the active compounds oxyphyllenone A (**3**) and magnodelavin C (**12**) and the target proteins IL-17 (PDB ID: 3VBC) and TNF (PDB ID: 5MU8). (**a**) Global binding conformations of the ligands localized within the respective active site pockets. (**b**) Three-dimensional (3D) visualizations of the optimal binding modes. (**c**) Two-dimensional (2D) schematic diagrams detailing the protein–ligand contact residues (hydrogen bonds are indicated by yellow dashed lines).

Guided by the network pharmacology results, molecular docking studies were conducted to explore the interactions between the active compounds (**3** and **12**) and the key inflammatory targets, IL-17 (PDB ID: 3VBC) and TNF (PDB ID: 5MU8). For docking with IL-17 (3VBC), compound **3** in its most stable conformation exhibited a binding free energy of −4.82 kcal/mol, forming four hydrogen bonds with residues Lys439, Lys388, Asp387, and His436, alongside hydrophobic interactions with Ala386, Ala479, and Leu437. Similarly, compound **12** yielded a binding affinity of −5.72 kcal/mol, establishing three hydrogen bonds with Lys388, Leu437, and Glu475, and engaging in hydrophobic contacts with Asp387, Lys478, Gln481, Ala479, and His438. Furthermore, in the simulation with TNF (5MU8), the lowest-energy pose of compound **3** displayed a binding energy of −6.07 kcal/mol. It was anchored by two hydrogen bonds with Gly121 and Leu120, and multiple hydrophobic interactions with Gly122, Ser60, Tyr59, Tyr119, Leu120, and Gln61. Compound **12** demonstrated an even stronger binding affinity of -6.94 kcal/mol with TNF, interacting via two hydrogen bonds (Ser60 and Leu120) and an extensive hydrophobic network involving Gly121, Gly122, Leu57, Leu120, Tyr59, Tyr119, Tyr151, and Gln61. The detailed docking modes are illustrated in Figure 10.

## 4. Discussion

Natural plant-derived antioxidants exhibit the advantages of multi-pathway synergistic regulation and low toxic side effects [25]. Compared with chemically synthesized drugs that act on a single target, they exert modulatory effects via multiple pathways with low toxicity. *C. migao*, a classic Miao ethnomedicine clinically used for ischemic heart diseases, is abundant in terpenoid antioxidants; however, its redox-regulating cardioprotective mechanisms have not yet been systematically elucidated [26]. Previous studies have demonstrated that the characteristic phenylpropanoids derived from *Cinnamomum cassia* and *Cinnamomum verum* exhibit significant cardioprotective properties. For instance, the traditional herb pair of Ramulus Cinnamomi and Radix Glycyrrhizae alleviates hypoxia-induced injury in H9c2 cardiomyocytes by regulating the PI3K/Akt signaling pathway [27]. Furthermore, cinnamaldehyde has been reported to mitigate acute myocardial infarction by regulating ferroptosis via the same PI3K/Akt pathway [28]. *C. migao*, recognized as one of the “Top Ten Miao Medicines” in Guizhou Province, China, has been extensively utilized in clinical practice for the prevention and treatment of coronary heart disease. Despite sharing the same genus (*Cinnamomum*, Lauraceae) with *C. cassia* and *C. verum*, *C. migao* possesses a distinctly different chemical profile. Specifically, the ethyl acetate fraction of *C. migao* is predominantly composed of sesquiterpenes and monoterpenes, with a notable absence of cinnamaldehyde. While extensive prior research has focused on the cardioprotective effects of phenylpropanoids (e.g., cinnamaldehyde) from *Cinnamomum* species, the present study broadens this perspective for the first time by shifting the focus to the characteristic sesquiterpenoids of *C. migao*. Herein, we evaluated the in vitro and in vivo protective effects of these sesquiterpenes against acute myocardial ischemia and injury, along with exploring their underlying mechanisms.

In the present study, we combined in vitro and in vivo experiments, network pharmacology, phytochemical isolation, and molecular docking to systematically explore the cardioprotective effects and underlying mechanisms of the ethyl acetate extract of *C. migao* (MGE). Preliminary screening using an OGD/R cardiomyocyte injury model confirmed that MGE serves as the core active fraction of *C. migao* against hypoxic injury, resolving the ambiguity regarding the localization of bioactive fractions of this medicinal herb. MGE markedly restored the viability of OGD/R-injured H9c2 cardiomyocytes, further verifying that MGE represents the primary pharmacologically active component of *C. migao*. Notably, H9c2 myoblasts are immature, non-terminally differentiated cardiomyocytes, which somewhat limits the physiological translational relevance of our in vitro findings. Future studies using primary mature cardiomyocytes will be conducted to further verify and consolidate the present cellular results. Via UPLC-MS/MS combined with the GNPS molecular networking platform, a total of 30 chemical constituents were systematically identified from MGE, establishing a comprehensive chemical profiling database for MGE. Distinct from previous studies that solely focused on its volatile essential oil, the present work directly links the medium-polarity sesquiterpenoid-enriched fraction to anti-acute myocardial ischemia activity, and confirms sesquiterpenoids as the core material basis responsible for the cardiovascular protective effects of *C. migao*. These findings provide an accurate material foundation for subsequent screening of bioactive monomers and the development of novel therapeutic agents. In vivo experiments based on the ISO-induced AMI rat model confirmed that MGE exerted dose-dependent cardioprotective effects, including alleviating ST-segment elevation on electrocardiograms, reducing serum levels of myocardial injury biomarkers CK-MB and LDH, and ameliorating pathological lesions such as disorganized myocardial fibers, cardiomyocyte edema, inflammatory infiltration and myocardial necrosis. The therapeutic efficacy of high-dose MGE was comparable to that of the positive control propranolol, further demonstrating the potent and stable pharmacological activity of MGE. Meanwhile, MGE exhibited dual antioxidant and anti-inflammatory properties. It markedly elevated the activities of antioxidant enzymes SOD and CAT and reduced the level of lipid peroxidation product MDA to restore redox homeostasis, while suppressing the secretion of pro-inflammatory cytokines TNF-α, IL-6 and IL-1β and blocking the amplified inflammatory cascade triggered by myocardial ischemia.

Existing studies have demonstrated that NOX4 acts as a key catalytic enzyme triggering massive ROS burst in ischemic myocardium. Excessive ROS persistently activates p38 MAPK, which further facilitates the nuclear translocation of NF-κB p65 to initiate the transcription of numerous downstream pro-inflammatory genes [14]. In parallel, the hypoxic microenvironment upregulates HIF-1α, which further exacerbates redox imbalance and inflammatory infiltration [29]. In contrast, the Nrf2/HO-1 pathway represents the central endogenous antioxidant defense system that eliminates excess ROS and interrupts inflammatory signal transduction [22]. To further dissect the molecular regulatory network underlying the synergistic antioxidant, anti-inflammatory and anti-hypoxic effects of MGE, this study integrated network pharmacology prediction and quantitative Western blot protein detection. The results confirmed that MGE alleviated AMI injury via bidirectional regulation of the HIF-1α/Nrf2/NF-κB/MAPK signaling axis. On one hand, MGE downregulated the protein expression of NOX4, HIF-1α, p38 MAPK and NF-κB p65, thereby suppressing the initiation of oxidative stress, hypoxic damage and inflammatory signaling at the source. On the other hand, MGE significantly promoted the nuclear translocation of Nrf2 and upregulated its downstream effector HO-1 to strengthen endogenous antioxidant defense, ultimately breaking the vicious pathological cycle of mutual promotion among oxidative stress, hypoxia and inflammation at the upstream regulatory level. A previous study reported that the anti-inflammatory efficacy of the Tongmai Yangxin Pill against coronary heart disease is tightly linked to the crosstalk between estrogen receptor and NF-κB signaling cascades, which supports our finding that modulation of the NF-κB pathway is a conserved anti-ischemia mechanism of cardioprotective herbal medicines [30]. Current Western blot data only reveal correlative pathway protein changes, failing to confirm causal regulation between MGE and the HIF-1α/Nrf2/NF-κB/MAPK axis claimed in the title. In follow-up research, we will conduct functional rescue experiments combining pharmacological intervention and genetic manipulation: pathway-specific inhibitors/activators and gene silencing will be applied in OGD/R H9c2 cells and ISO-induced AMI rats. We will assess whether blocking core pathway nodes abolishes MGE’s anti-oxidative, anti-inflammatory and cardioprotective phenotypes, so as to confirm the indispensable causal role of this signaling cascade in mediating MGE’s anti-AMI efficacy.

To identify the characteristic monomers contributing to the core therapeutic efficacy of MGE, 12 sesquiterpenoid monomers were isolated from the bioactive fraction and subjected to in vitro cellular activity screening in this study. Five monomers were found to significantly reverse the OGD/R-induced decline in cardiomyocyte viability, among which oxyphyllenone A and magnodelavin C exhibited the most potent cytoprotective effects and were identified as the key pharmacodynamic markers in MGE. KEGG enrichment analysis from network pharmacology indicated that the IL-17 and TNF inflammatory signaling pathways were the top enriched pathways. Further molecular docking assays verified that these two pivotal sesquiterpenoids could stably bind to the core inflammatory targets IL-17 and TNF with high binding affinities. They occupied the active pockets of target proteins via hydrogen bonds and hydrophobic interactions, thereby directly blocking the transduction of pro-inflammatory signals. These findings formed a closed-loop evidence chain covering “crude extract-bioactive fraction-characteristic monomer-core target”. It should be critically noted that the network pharmacology and molecular docking analysis adopted in this study are purely in silico predictive tools, which cannot independently serve as direct experimental evidence to verify the causal regulatory relationship between MGE and the HIF-1α/Nrf2/NF-κB/MAPK signaling pathway. First, network pharmacology prediction relies on public bioinformatics databases with inherent bias, including incomplete target annotation of natural sesquiterpenoids, inconsistent disease target screening standards, and ignorance of compound absorption, metabolism and bioavailability in vivo; the predicted compound–target interactions only reflect potential binding possibilities without confirming actual intracellular regulatory effects [31]. Second, molecular docking merely simulates static binding affinity between small-molecule monomers and target protein crystal structures under ideal in vitro conditions, failing to recapitulate the dynamic intracellular microenvironment, protein post-translational modification, and multi-component synergistic effects of MGE extracts [32]. For these reasons, we treated all computational predictions only as directional screening clues rather than conclusive mechanistic evidence in this work. Furthermore, our follow-up accepted independent research will utilize specific pharmacological inhibitors and activators of HIF-1α, Nrf2, NF-κB and p38 MAPK to conduct functional rescue experiments, which will supply definitive experimental proof to validate the regulatory axis preliminarily screened by computational analysis.

Unlike most previous studies that merely evaluated the overall activity of crude herbal extracts, the present work accurately identified the core bioactive constituents of *C. migao* against myocardial ischemia. These findings provide direct experimental support for the subsequent establishment of quality control indicators and the development of lead monomer compounds. As a low-toxicity natural multi-target agent with prominent antioxidant and anti-inflammatory capacities, MGE effectively addresses the limitations of conventional clinical AMI drugs, including insufficient reperfusion efficacy, bleeding risks, hepatorenal toxicity, and single-target therapeutic limitations [13]. It lays a solid experimental foundation for developing safe, affordable, multi-target novel ethnic medicinal preparations for the prevention and treatment of myocardial ischemia.

## 5. Conclusions

In conclusion, the present study demonstrates the potent cardioprotective efficacy of MGE against AMI. Phytochemical profiling confirmed that MGE is rich in sesquiterpenoids, which constitute its principal bioactive components. Mechanistically, MGE alleviates myocardial ischemic injury by quenching excessive oxidative stress and blocking subsequently hypoxic and inflammatory cascades via comprehensive modulation of the oxidative stress-related HIF-1α/Nrf2/NF-κB/MAPK signaling axis. Among the isolated monomers, Oxyphyllenone A and magnodelavin C are identified as the core bioactive constituents of MGE. These findings clarify the modern pharmacological basis for the traditional application of *C. migao* in cardiovascular disease intervention and highlight its potential as a novel, safe, and multi-target natural candidate for the prevention and treatment of AMI.

## Data Availability

The data presented in this study are available on request from the corresponding author.

## Supplementary Materials

The following supporting information can be downloaded at: https://www.mdpi.com/article/10.3390/antiox15080970/s1, Figure S1: Chemical components in MGE.; Figure S2: Structures of the isolated compounds from *C. migao* and their in vitro anti-AMI effects. Table S1: Compounds of MGE.

## References

[1] (2026). GBD 2023 IHD & Dietary Risk Factors Collaborators. Global, regional and national burden of ischemic heart disease attributable to suboptimal diet, 1990–2023: A Global Burden of Disease study. Nat. Med..

[2] Kim J.A., Kim S.Y., Virk H.U.H., Alam M., Sharma S., Johnson M.R., Krittanawong C. (2026). Acute myocardial infarction in pregnancy. Cardiol. Rev..

[3] Udell J.A., Bahit M.C., Campbell P., Butler J., Chopra V.K., Bayes-Genis A., Bozkurt B., Petrie M.C., Vaduganathan M. (2025). Prevention of heart failure after acute myocardial infarction. Lancet.

[4] Saito Y., Oyama K., Tsujita K., Yasuda S., Kobayashi Y. (2023). Treatment strategies of acute myocardial infarction: Updates on revascularization, pharmacological therapy, and beyond. J. Cardiol..

[5] Wilkinson-Stokes M., Betson J., Sawyer S. (2023). Adverse events from nitrate administration during right ventricular myocardial infarction: A systematic review and meta-analysis. Emerg. Med. J..

[6] Lombardi M., Boivin-Proulx L.A., Jeronimo A., Mejia-Renteria H., Gonzalo N., Gori T., Mehran R., Escaned J. (2026). Angina after percutaneous coronary interventions. Eur. Heart J..

[7] Pereira N.L., Farkouh M.E., So D., Lennon R., Geller N., Mathew V., Bell M., Bae J.H., Jeong M.H., Chavez I. (2020). Effect of genotype-guided oral P2Y12 inhibitor selection vs conventional clopidogrel therapy on ischemic outcomes after percutaneous coronary intervention: The TAILOR-PCI randomized clinical trial. JAMA.

[8] Proctor P., Leesar M.A., Chatterjee A. (2018). Thrombolytic therapy in the current ERA: Myocardial infarction and beyond. Curr. Pharm. Des..

[9] Rousan T.A., Mathew S.T., Thadani U. (2016). The risk of cardiovascular side effects with anti-anginal drugs. Expert Opin. Drug Saf..

[10] Boden W.E., Marzilli M., Crea F., Mancini G.B.J., Weintraub W.S., Taqueti V.R., Pepine C.J., Escaned J., Al-Lamee R., Gowdak L.H.W. (2023). Evolving management paradigm for stable ischemic heart disease patients: JACC review topic of the week. J. Am. Coll. Cardiol..

[11] Corradi F., Masini G., Bucciarelli T., Caterina R.D. (2023). Iron deficiency in myocardial ischaemia: Molecular mechanisms and therapeutic perspectives. Cardiovasc. Res..

[12] Jiang Q., Chen X., Gong K., Xu Z., Chen L., Zhang F. (2025). M6a demethylase FTO regulates the oxidative stress, mitochondrial biogenesis of cardiomyocytes and PGC-1a stability in myocardial ischemia-reperfusion injury. Redox Rep..

[13] Xiang Q., Yi X., Zhu X.-H., Wei X., Jiang D.-S. (2024). Regulated cell death in myocardial ischemia-reperfusion injury. Trends Endocrinol. Metab..

[14] Zhang S., Yan F., Luan F., Chai Y., Li N., Wang Y.W., Chen Z.L., Xu D.Q., Tang Y.P. (2024). The pathological mechanisms and potential therapeutic drugs for myocardial ischemia reperfusion injury. Phytomedicine.

[15] Bugger H., Pfeil K. (2020). Mitochondrial ROS in myocardial ischemia reperfusion and remodeling. Biochim. Biophys. Acta Mol. Basis. Dis..

[16] Jin S., Kang P.M. (2024). A Systematic Review on Advances in Management of Oxidative Stress-Associated Cardiovascular Diseases. Antioxidants.

[17] Toldo S., Abbate A. (2024). The role of the NLRP3 inflammasome and pyroptosis in cardiovascular diseases. Nat. Rev. Cardiol..

[18] Chen J., Huang X., Tong B., Wang D., Liu J., Liao X., Sun Q. (2021). Effects of rhizosphere fungi on the chemical composition of fruits of the medicinal plant *Cinnamomum migao* endemic to southwestern China. BMC Microbiol..

[19] Zhou L., Yang L.S., Wang L., Liu H.D., Gao M., Chen F.J., Yang J., Li Q.J., Yang X.S. (2023). Cinnamigones A-C, three highly oxidized guaiane-type sesquiterpenes with neuroprotective activity from *Cinnamomum migao*. Phytochemistry.

[20] Muhammad I., Luo W., Shoaib R.M., Li G.L., Hassan S.S.U., Yang Z.H., Xiao X., Tu G.L., Yan S.K., Ma X.P. (2021). Guaiane-type sesquiterpenoids from *Cinnamomum migao* H. W. Li: And their anti-inflammatory activities. Phytochemistry.

[21] Xiao Y., Muhammad I., Ma X., Yu H., Yan S., Xiao X., Jin H. (2022). Camganoids A and B, two new sesquiterpenes with different carbon skeletons isolated from fruits of *Cinnamomum migao*. Chin. Herb. Med..

[22] Muhammad I., Hassan S.S.U., Xu W.J., Tu G.L., Yu H.J., Xiao X., Yan S.K., Jin H.Z., Bungau S. (2023). An extensive pharmacological evaluation of novel anti-nociceptive and IL-6 targeted anti-inflammatory guaiane-type sesquiterpenoids from *Cinnamomum migao* H. W. Li through in-depth in-vitro, ADMET, and molecular docking studies. Biomed. Pharmacother..

[23] Zhou L., Pan X., Chen F.J., Peng M., Wang Y., Gao M., Yang L.S., Li L.Q., Yang J., Yang X.S. (2025). Targeted discovery of oxygen-bridged sesquiterpenes from *Cinnamomum migao* by MS/MS-based molecular networking and their neuroprotective activities. Phytochemistry.

[24] Zhou L., Chen F., Yang L., Peng M., Pan X., Lou H., Yang J., Yang X., Li Q. (2024). Vicinal diol sesquiterpenes from *Cinnamomum migao* with neuroprotective effects in PC12 cells. Int. J. Mol. Sci..

[25] Verma V.K., Bhardwaj P., Prajapati V., Bhatia A., Purkait S., Arya D.S. (2024). Flavonoids as therapeutics for myocardial ischemia-reperfusion injury: A comprehensive review on preclinical studies. Lab. Anim. Res..

[26] Arshad M.T., Ali M.K.M., Maqsood S., Ikram A., Hossain M.S., Aljameel A.I., Al-Farga A., Gnedeka K.T. (2025). Dietary Phytochemicals in Cardiovascular Disease Prevention and Management: A Comprehensive Review. Food Sci. Nutr..

[27] Xu F., Wang Q., Li R., Adu-Frimpong M., Zhu L., Miao X., Liu H., Chen Y., Wang Q., Tong S. (2025). Ramulus Cinnamomi (*Cinnamomum cassia*)-Radix Glycyrrhizae (*Glycyrrhiza uralensis*) herb treat hypoxia-induced injury H9c2 cells by modulating the PI3K/Akt pathway. Fitoterapia.

[28] Yang M., Lin D.M., He L.M., Shi X.X., Wang Y.C., Jin Y., Huang S.W. (2025). Cinnamaldehyde mitigates acute myocardial infarction by regulating ferroptosis through the PI3K-AKT signaling pathway. Int. Immunopharmacol..

[29] Packer M. (2020). Mutual Antagonism of Hypoxia-Inducible Factor Isoforms in Cardiac, Vascular, and Renal Disorders. JACC Basic Transl. Sci..

[30] Fan Y., Liu J., Miao J., Zhang X., Yan Y., Bai L., Chang J., Wang Y., Wang L., Bian Y. (2021). Anti-inflammatory activity of the Tongmai Yangxin pill in the treatment of coronary heart disease is associated with estrogen receptor and NF-kappaB signaling pathway. J. Ethnopharmacol..

[31] Merecz-Sadowska A., Sadowski A., Zielińska-Bliźniewska H., Zajdel K., Zajdel R. (2025). Network Pharmacology as a Tool to Investigate the Antioxidant and Anti-Inflammatory Potential of Plant Secondary Metabolites-A Review and Perspectives. Int. J. Mol. Sci..

[32] Rigby S.P. (2024). Uses of Molecular Docking Simulations in Elucidating Synergistic, Additive, and/or Multi-Target (SAM) Effects of Herbal Medicines. Molecules.

