# Supplementary material for: Cinnamomum migao Active Extracts Ameliorate Acute Myocardial Ischemia via Modulation of the HIF-1α/Nrf2/NF-κB/MAPK Pathway Downstream of Oxidative Stress
Source: Antioxidants (Basel). 2026 Aug 5;15(8):970. doi: 10.3390/antiox15080970 (PMC13509234; doi:10.3390/antiox15080970)
Supplement: Supplementary file 1 [file antioxidants-15-00970-s001.zip › antioxidants-4423568-supplementary.pdf]

# Supplementary material

**1. Plant material**

The mature fruits of *C. migao* were collected from Luodian County, Guizhou Province, China. The plant material was authenticated by Dr. Jing-Zhong Chen (Guizhou University of Traditional Chinese Medicine). A voucher specimen (GZLD20251011) has been deposited at the Natural Products Research Center of Guizhou Province.

**2. LC−MS/MS analysis**

Liquid chromatography-tandem mass spectrometry (LC-MS/MS) analysis of the *C. migao* subfractions was performed on a Dionex Ultimate 3000 RSLC system coupled with a heated electrospray ionization (HESI-II) dual source. Chromatographic separation was achieved utilizing a Waters Acquity UPLC HSS T3 column (100 × 2.1 mm, 1.8 μm). The mobile phase consisted of 0.1% aqueous formic acid (v/v, solvent B) and acetonitrile containing 0.1% formic acid (solvent C). Elution was carried out at a constant flow rate of 0.3 mL/min with the following gradient profile: an initial hold at 5% C for 2 min, a linear increase to 95% C from 2 to 42 min, an isocratic phase at 95% C until 47 min, and a final re-equilibration step at 5% C (47.1–50 min).

**3 Molecular docking of active constituents**

Molecular docking simulations were conducted using AutoDock 4 software to investigate the binding modes of compounds **3** and **12** with key inflammatory targets. The 3D crystal structures of the target proteins, IL-17 (PDB ID: 3VBC) and TNF (PDB ID: 5MU8), were retrieved from the RCSB Protein Data Bank (https://www.rcsb.org/). Concurrently, the 3D geometries of compounds **3** and **12** were generated utilizing Chem3D 22.0 software. Following the docking procedures, the conformations with the optimal docking scores were visualized and analyzed using PyMOL 2.4. Furthermore, two-dimensional (2D) diagrams illustrating the protein-ligand interactions were generated via the LigPlot$^+$ Molecular Graphics System (version 2.3.1).

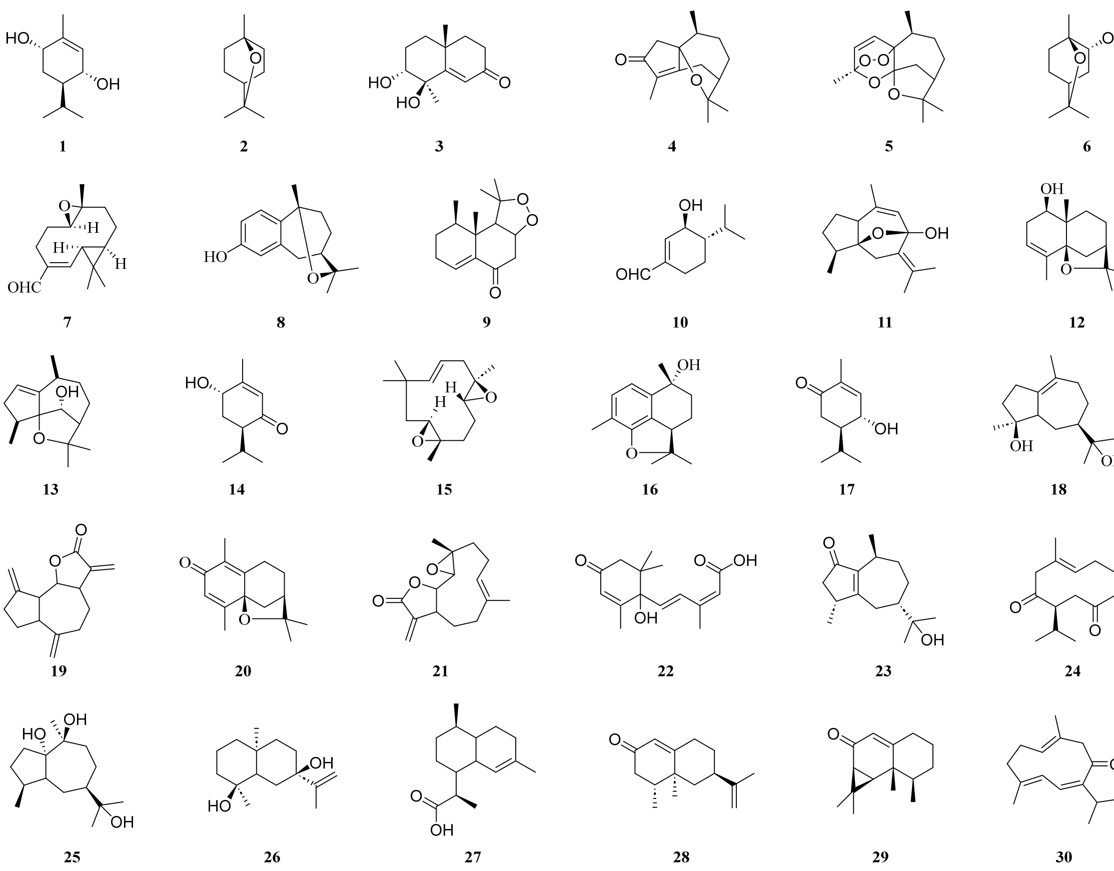

**Figure S1.** Chemical components in MGE.

**NMR data of isolation compounds**

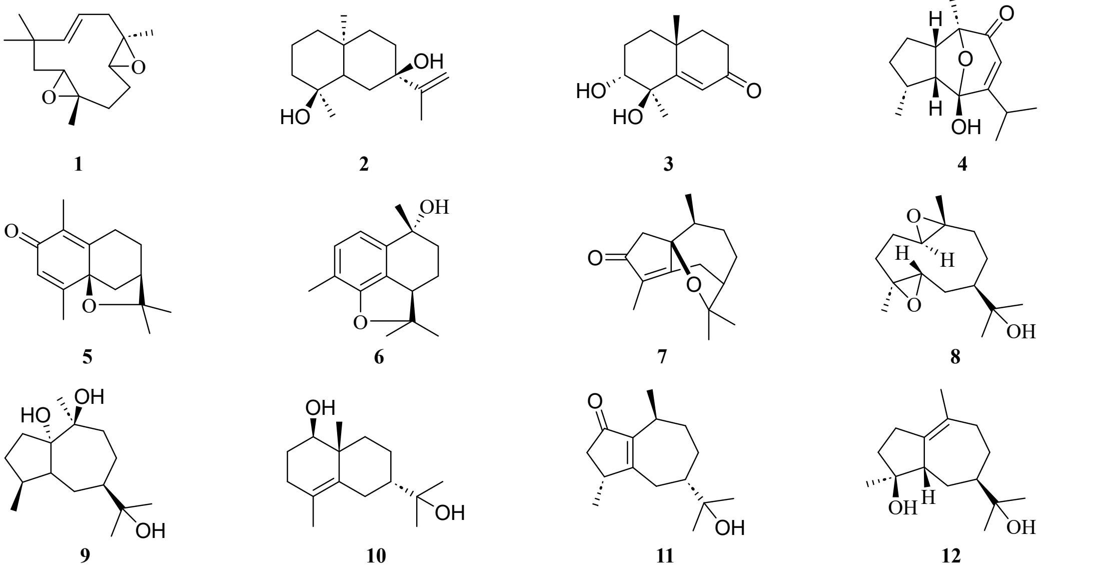

**Figure S2. Structures of the isolated compounds from *C. migao* and their in vitro anti-AMI effects.**

Humulene diepoxide A (**1**). ESI-MS (*m/z*): 259.2 $[M + Na]^+$, $C_{15}H_{24}O_2$. $^1$H-NMR (600 MHz, $CDCl_3$) $\delta_H$ 2.48 (1H, d, $J = 9.5$ Hz, H-2), 1.38(1H, m, H-3a), 1.61 (1H, m, H-3b), 5.31 (1H, d, $J = 15.6$ Hz, H-5), 5.49 (1H, ddd, $J = 15.6$, 10.6, 5.0 Hz, H-6), 1.65

(1H, m, H-7a), 2.61 (H, m, H-7b), 2.73 (1H, dd, $J$ = 10.1, 5.1 Hz, H-9), 1.38 (1H, m, H-10a), 2.21 (1H, m, H-10b), 1.08 (1H, m, H-11a), 2.13 (1H, m, H-11b), 1.31 (3H, s, Me-12), 1.20 (3H, s, Me-13), 1.08 (3H, s, Me-14), 1.31 (3H, s, Me-15); $^{13}$C-NMR(150 MHz, $CDCl_3$) $\delta_C$: 60.2 (C-1), 64.8 (C-2), 38.7 (C-3), 35.8 (C-4), 143.0 (C-5), 122.7 (C-6), 43.4 (C-7), 60.2 (C-8), 60.5 (C-9), 25.3 (C-10), 35.0 (C-11), 16.6 (C-12), 23.5 (C-13), 30.8 (C-14), 16.6 (C-15).

Teucdiol A (**2**). ESI-MS m/z: 238 $[M]^+$, $C_{15}H_{26}O_2$。$^1$H-NMR (400 MHz, $CDCl_3$) $\delta_H$ 4.82 (1H, br s, H-12a), 4.71 (1H, br s, H-12b), 0.89 (3H, s, Me-13), 1.01(3H, s, Me-14), 1.80 (3H, s, Me-15); $^{13}$C-NMR (150 MHz, $CDCl_3$) $\delta_C$: 42.1 (C-1), 21.1 (C-2), 44.3 (C-3), 74.5 (C-4), 49.3 (C-5), 41.6 (C-6), 72.7 (C-7), 32.1 (C-8), 31.8 (C-9), 35.5 (C-10), 153.9 (C-11), 109.2 (C-12), 19.4 (C-13), 18.3 (C-14), 22.5 (C-15).

Oxyphyllenone A (**3**). ESI-MS (*m/z*): 233 $[M + Na]^+$, $C_{12}H_{18}O_3$. $^1$H-NMR (400 MHz, $CDCl_3$) $\delta_H$ 1.44 (1H, m, H-1a), 1.84 (1H, td, $J$ = 13.8, 4.1 Hz, H-1b), 1.58 (1H, ddd, $J$ = 13.9, 6.9, 3.6 Hz, H-2a), 2.40 (1H, tdd, $J$ = 14.1, 4.1, 2.6 Hz, H-2b), 3.70 (1H, s, H-3), 6.02 (3H, s, H-6), 2.33 (1H, ddd, $J$ = 17.6, 3.4, 2.5Hz, H-8a), 2.64 (1H, ddd, $J$ = 17.6, 15.2, 5.0 Hz H-8b), 1.75 (1H, ddd, $J$ = 13.2, 5.0, 2.3 Hz, H-9a), 1.91 (1H, m, H-9b), 1.43, 1.48 (6H, s, Me-14, 15); $^{13}$C-NMR (150 MHz, $CDCl_3$) $\delta_C$: 35.3 (C-1), 25.4 (C-2), 76.2 (C-3), 73.9 (C-4), 172.6 (C-5), 126.1 (C-6), 203.4 (C-7), 34.9 (C-8), 41.0 (C-9), 25.5 (C-14), 25.6 (C-15).

Bulnesol (**4**). ESI-MS (*m/z*): 273 $[M + Na]^+$, $C_{15}H_{22}O_3$. $^1$H-NMR (600 MHz, $CDCl_3$) $\delta_H$ 2.91 (1H, m, H-1), 2.73 (1H, m, H-5), 5.92 (1H, s, H-8), 1.25 (3H, d, $J$ = 6.6 Hz, H-12), 1.18 (3H, d, $J$ = 6.9 Hz, H-12), 1.10 (3H, d, $J$ = 7.3 Hz, H-14), 1.48 (3H, s, H-15); $^{13}$C-NMR(150 MHz, $CDCl_3$) $\delta_C$: 52.6 (C-1), 33.1 (C-2), 24.7 (C-3), 36.5(C-4), 63.7 (C-5), 105.4 (C-6), 176.0 (C-7), 123.8 (C-8), 199.2 (C-9), 88.4 (C-10), 30.2 (C-11), 20.9 (C-12), 20.7 (C-13), 25.8 (C-14), 14.6 (C-15).

Cinnamigone H (**5**). HR-ESI-MS (*m/z*) 255.1348 $[M+Na]^+$, $C_{15}H_{20}O_2$. $^1$H-NMR (600 MHz, $CDCl_3$) $\delta_H$ 6.01 (1H, s, H-3), 2.59 (1H, dd, $J$ = 12.2, 6.2 Hz, H-6a), 1.71 (1H, d, $J$ = 12.2 Hz, H-6b), 2.22 (1H, m, H-7), 1.95 (1H, m, H-8a), 1.65 (1H, m, H-8b), 3.10 (1H, m, H-9a), 2.18 (1H, m, H-9b), 1.44 (3H, s, H-12), 1.30 (3H, s, H-12), 2.01 (3H, s, H-14), 2.23 (3H, s, H-15); $^{13}$C-NMR(150 MHz, $CDCl_3$) $\delta_C$: 148.4 (C-1), 194.6 (C-2),

134.7 (C-3), 169.1 (C-4), 88.2 (C-5), 41.4 (C-6), 45.5 (C-7), 27.6 (C-8), 35.6 (C-9), 138.6 (C-10), 84.0 (C-11), 23.1 (C-12), 30.9 (C-13), 13.4 (C-14), 21.1 (C-15).

Cinnamigone F (**6**). HR-ESI-MS (*m/z*) 255.1352 $[M + Na]^+$, $C_{15}H_{20}O_2$. $^1$H-NMR (600 MHz, $CDCl_3$) $\delta_H$ 6.89 (1H, d, *J* = 7.8 Hz, H-1), 6.96 (1H, d, *J* = 7.8 Hz, H-2), 2.99 (1H, dd, *J* = 12.1, 4.6 Hz, H-6), 1.77 (1H, m, H-7a), 1.63 (1H, m, H-7b), 2.14 (1H, m, H-8a), 1.79 (1H, m, H-8b), 1.11 (3H, s, H-12), 1.64 (3H, s, H-13), 2.16 (3H, s, H-14), 1.61 (3H, s, H-15); $^{13}$C-NMR(150 MHz, $CDCl_3$) $\delta_C$: 117.6 (C-1), 129.6 (C-2), 119.1 (C-3), 155.1 (C-4), 128.4 (C-5), 49.5 (C-6), 20.7 (C-7), 40.1 (C-8), 68.9 (C-9), 137.3 (C-10), 92.2 (C-11), 22.1 (C-12), 27.8 (C-13), 15.0 (C-14), 29.0 (C-15).

Cinnamigone C (**7**). HR-ESI-MS (*m/z*) 257.1510 $[M+Na]^+$, $C_{15}H_{22}O_2$. $^1$H-NMR (600 MHz, $CDCl_3$) $\delta_H$ 2.47 (1H, d, *J* = 17.5 Hz, H-2a), 2.36 (1H, d, *J* = 17.5 Hz, H-2b), 2.88 (1H, dd, *J* = 19.6, 5.7 Hz, H-6a), 2.57 (1H, dq, *J* = 19.6, 1.6 Hz, H-6a), 1.62 (1H, m, H-7), 2.16 (1H, m, H-8a), 1.63 (1H, m, H-8b), 1.68 (1H, m, H-9a), 1.33 (1H, m, H-9b), 1.92 (1H, m, H-10), 1.14 (3H, s, H-12), 1.30 (3H, s, H-13), 1.71 (3H, s, H-14), 1.05 (3H, d, *J* = 6.9 Hz, H-15); $^{13}$C-NMR(150 MHz, $CDCl_3$) $\delta_C$: 81.2 (C-1), 51.8 (C-2), 205.3 (C-3), 135.6 (C-4), 171.2 (C-5), 25.6 (C-6), 43.9 (C-7), 23.5 (C-8), 27.4 (C-9), 38.3 (C-10), 74.8 (C-11), 28.9 (C-12), 27.9 (C-13), 7.9 (C-14), 16.4 (C-15).

Cinnamigone D (**8**). HR-ESI-MS (*m/z*) 255.1950 $[M+H]^+$, $C_{15}H_{26}O_3$. $^1$H-NMR (600 MHz, $CDCl_3$) $\delta_H$ 3.09 (1H, dd, *J* = 9.2, 5.9 Hz, H-1), 2.32 (1H, m, H-2a), 1.50 (1H, m, H-2b), 2.18 (1H, m, H-3a), 1.22 (1H, m, H-3b), 2.90 (1H, dd, *J* = 10.4, 4.3 Hz, H-5), 1.82 (1H, m, H-6a), 1.48 (1H, m, H-6a), 1.39 (1H, m, H-7), 1.84 (1H, m, H-8a), 1.36 (1H, m, H-8b), 1.87 (1H, m, H-9a), 1.05 (1H, m, H-9b), 1.17 (3H, s, H-12), 1.19 (3H, s, H-13), 1.39 (3H, s, H-14), 1.33 (3H, s, H-15); $^{13}$C-NMR(150 MHz, $CDCl_3$) $\delta_C$: 60.4 (C-1), 24.8 (C-2), 34.6 (C-3), 59.6 (C-4), 63.8 (C-5), 29.9 (C-6), 48.2 (C-7), 25.1 (C-8), 41.6 (C-9), 60.1 (C-10), 73.7 (C-11), 26.6 (C-12), 26.9 (C-13), 15.7 (C-14), 16.9 (C-15).

Stachytriol (**9**). ESI-MS (m/z): 279 $[M + Na]^+$, $C_{15}H_{28}O_3$。 $^1$H-NMR (600 MHz, $CDCl_3$) $\delta_H$ 1.33 (1H, m, H-2a), 2.41 (1H, m, H-2b), 1.39 (1H, m, H-3a), 1.88 (1H, m, H-3b), 2.55 (1H, m, H-4), 1.88 (1H, m, H-5), 1.45 (1H, m, H-6a), 1.57 (1H, m, H-6b), 1.79 (1H, m, H-7), 1.63 (1H, m, H-8a), 2.02 (1H, m, H-8b), 1.63 (1H, m, H-9a), 2.02

(1H, m, H-9b), 1.19 (3H, s, Me-12), 1.30 (3H, s, Me-13), 0.89 (3H, d, $J$ = 6.9 Hz, Me-14), 1.11 (3H, s, Me-15); $^{13}$C-NMR(150 MHz，CDCl$_3$) $\delta_C$: 90.7 (C-1), 32.4 (C-2), 29.6 (C-3), 35.4 (C-4), 47.9 (C-5), 24.5 (C-6), 36.5 (C-7), 19.3 (C-8), 26.2 (C-9), 75.8 (C-10), 73.7 (C-11), 29.2 (C-12), 29.4 (C-13), 15.1 (C-14), 26.8 (C-15).

7-Epi-4-eudesmene-l$\beta$,11-diol (**10**). ESI-MS (m/z): 251 [M + Na]$^+$, $C_{15}H_{26}O_2$. $^1$H-NMR (600 MHz, CDCl$_3$) $\delta_H$ 3.51 (1H, d, $J$ = 8.8 Hz, H-1), 2.03 (1H, m, H-6b), 2.61 (1H, dd, $J$ = 15.3, 3.5 Hz, H-6b), 1.17 (3H, s, Me-12), 1.23 (3H, s, Me-13), 1.63 (3H, s, Me-14)，1.02 (3H, s, Me-15); $^{13}$C-NMR(150 MHz, CDCl$_3$) $\delta_C$: 75.5 (C-1), 27.2 (C-2), 31.5 (C-3), 125.3 (C-4), 133.8 (C-5), 25.3 (C-6), 44.2 (C-7), 22.0 (C-8), 33.6 (C-9), 39.6 (C-10), 74.3 (C-11), 27.5 (C-12), 29.6 (C-13), 19.3 (C-14), 19.5 (C-15).

Lancilimbnoid D (**11**). ESI-MS (*m/z*): 259 [M+Na]$^+$, $C_{15}H_{24}O_2$. $^1$H-NMR (600 MHz, CDCl$_3$) $\delta_H$ 1.97 (1H, m, H-3a), 2.56 (1H, dd, $J$ = 18.5, 6.3 Hz, H-3b), 2.72 (1H, m, H-4), 2.26 (1H, dd, $J$ = 16.3, 10.2Hz, H-6a), 2.70 (1H, m, H-6b), 1.86 (1H, m, H-7), 1.42 (1H, m, H-8a), 1.92 (1H, m, H-8b), 1.67 (1H, m, H-9a), 1.86 (1H, m, H-9b), 2.72 (1H, m, H-10), 1.25 (3H, s, Me-12), 1.18 (3H, s, Me-13), 1.19 (3H, d, $J$ = 7.2 Hz, Me-14), 1.10 (3H, d, $J$ = 7.2 Hz, Me-15); $^{13}$C-NMR(150 MHz，CDCl$_3$) $\delta_C$: 144.8 (C-1), 208.7 (C-2), 47.3 (C-3), 37.1(C-4), 178.8 (C-5), 31.5 (C-6), 48.7 (C-7), 27.0 (C-8), 33.5 (C-9), 27.5 (C-10), 73.6 (C-11), 28.7 (C-12), 25.3 (C-13), 19.6 (C-14), 18.2 (C-15).

Magnodelavin C (**12**). ESI-MS (*m/z*): 261 [M + Na]$^+$, $C_{15}H_{26}O_2$. $^1$H-NMR (600 MHz, CDCl$_3$) $\delta_H$ 2.16 (1H, m, H-2a), 2.27 (1H, m, H-2b), 1.68 (2H, m, $H_2$-3), 2.61 (1H, m, H-5), 1.54 (1H, m, H-6a), 1.84 (1H, m, H-6b), 1.72 (1H, m, H-7), 1.63 (1H, m, H-8a), 1.73 (1H, m, H-8b), 2.13 (1H, m, H-9a), 2.29 (1H, m, H-9b), 1.18, 1.16 (6H, s, Me-12,13), 1.03 (3H, s, Me-14), 1.50 (3H, s, Me-15); $^{13}$C-NMR(150 MHz，CDCl$_3$) $\delta_C$: 135.7 (C-1), 28.4 (C-2), 39.1 (C-3), 80.9 (C-4), 49.2 (C-5), 27.8 (C-6), 46.4 (C-7), 25.6 (C-8), 34.6 (C-9), 127.9 (C-10), 74.3 (C-11), 28.1 (C-12), 26.6 (C-13), 22.0 (C-14), 21.0 (C-15).

**Table S1.** Compounds of MGE.

| Peak | Compound name | RT (min) | Adduction | Molecular Ion (*m/z*) | Formular | MS/MS |
|---|---|---|---|---|---|---|
| **1** | (3*R*, 4*R*, 6*S*)-3, 6-dihydroxy-1- menthene | 1.99 | $[M-H]^-$ | 169.0132 | $C_{10}H_{18}O_2$ | 169.0133; 136.5271; 125.0232; 107.0127; 97.0281; 81.0332; 69.0332 |
| **2** | 1, 8-Cineole | 4.04 | $[M-H]^-$ | 153.0182 | $C_{10}H_{18}O$ | 153.0182; 123.0439; 109.0282 |
| **3** | Oxyphyllenone A | 11.24 | $[M-H]^-$ | 209.0448 | $C_{12}H_{18}O_3$ | 209.0812; 191.0342; 165.0547; 149.0597; 123.0441; 106.0411; 59.0125 |
| **4** | Cinnamigone C | 12.74 | $[M+H]^+$ | 236.1691 | $C_{15}H_{22}O_2$ | 222.7169; 123.9667; 105.0701; 91.0548; 79.0545; 59.0500 |
| **5** | Cinnamigone A | 12.84 | $[M+H]^+$ | 267.1584 | $C_{15}H_{22}O_4$ | 249.7361; 119.0853, 105.0702, 91.0546, 79.0547, 67.05490, 61.4616 |
| **6** | (1*S*, 2*S*, 4*R*)-*trans*-2-hydroxy-1, 8-cineole | 13.31 | $[M-H]^-$ | 169.0132 | $C_{10}H_{18}O_2$ | 169.0860; 139.0023; 123.0802; 111.0075; 97.0279; 73.4920 |
| **7** | madolins A | 13.70 | $[M+H]^+$ | 235.1692 | $C_{15}H_{22}O_2$ | 234.3661; 218.2905; 159.9593; 117.0706; 105.0704; 93.0702; 79.0549; 77.0391; 65.0394 |
| **8** | Cinnamigone E | 14.01 | $[M+H]^+$ | 233.0629 | $C_{15}H_{22}O_2$ | 217.2025; 165.7296; 157.7066; 142.3548; 123.1961; 105.0705; 80.4341; 73.8721 |
| **9** | Nardosinone | 14.46 | $[M+H]^+$ | 251.1626 | $C_{15}H_{22}O_3$ | 225.4222; 209.3460; 173.1324; 157.1006; 131.0851; 105.0699; 91.0544; 79.0544; 67.0547 |
| **10** | (−)-Eucamalol | 14.79 | $[M+H]^+$ | 169.0493 | $C_{10}H_{16}O_2$ | 169.1220; 151.1116; 123.1168; 109.0651; 81.0704; 69.0705 |
| **11** | Curcumenol | 15.10 | $[M+H]^+$ | 235.1317 | $C_{15}H_{22}O_2$ | 235.1685; 194.1550; 159.1167; 133.1010; 119.0853; 105.0699; 91.0544; 81.0702; 67.0548 |
| **12** | Cinnamigone G | 15.34 | $[M+H]^+$ | 237.1835 | $C_{15}H_{24}O_2$ | 237.1845; 216.9163; 209.6120; 187.9192; 145.1004; 133.6577; 121.1006; 107.0855; 81.0701; 67.0548; 55.0549 |
| **13** | Cinnamigone B | 15.99 | $[M+H]^+$ | 237.1848 | $C_{15}H_{24}O_2$ | 222.9867; 208.2826; 109.0763; 95.0858; 79.05479; 71.0498; 67.0549; 55.0550 |
| **14** | (4*S*, 6*R*)-4-Hydroxy-3-methyl-6-(1- | 16.09 | $[M+H]^+$ | 169.0493 | $C_{10}H_{16}O_2$ | 169.1219; 151.1115; 123.1168; 107.0857; 81.0704; 67.0549 |

| | | | | | | |
|---|---|---|---|---|---|---|
| | methylethyl)-2-cyclohexen-1-one | | | | | |
| **15** | Humulene diepoxide A | 16.11 | $[M+H]^+$ | 237.1835 | $C_{15}H_{24}O_2$ | 237.1854; 219.7179; 201.3285; 179.1430; 159.1160; 151.1113; 145.1012; 119.0854; 107.0852; 95.0855; 81.0701; 67.0548; 55.0548 |
| **16** | Cinnamigone F | 16.55 | $[M+H]^+$ | 233.0629 | $C_{15}H_{20}O_2$ | 215.7584; 190.5279; 165.3879; 126.1305; 120.7208; 116.0411; 84.2879; 60.8492 |
| **17** | (4*R*,5*S*)-4-hydroxy-5-isopropyl-2-methylcyclohex-2-en-1-one | 16.89 | $[M+H]^+$ | 169.0493 | $C_{10}H_{16}O_2$ | 169.0858; 137.0596; 109.0651; 91.0547; 81.0704 |
| **18** | Magnodelavin C | 19.44 | $[M+Na]^+$ | 251.1637 | $C_{15}H_{26}O_2$ | 251.1635; 233.1531; 191.1426; 173.1321; 153.0907; 127.0753; 107.0857 |
| **19** | Dehydrocostus lactone | 20.48 | $[M-H]^-$ | 229.1217 | $C_{15}H_{18}O_2$ | 181.5809; 131.4202; 112.5499; 69.3367; 60.6503; 51.8575 |
| **20** | Cinnamigone H | 20.67 | $[M+H]^+$ | 233.0629 | $C_{15}H_{22}O_2$ | 211.1242; 192.5465; 176.7299; 167.0607; 142.9526; 116.0541; 97.2967; 87.8473; 77.0392; 67.6377 |
| **21** | Parthenolide | 20.86 | $[M+H]^+$ | 249.1470 | $C_{15}H_{20}O_3$ | 231.1379; 189.1263; 171.1155; 145.1005; 119.0851; 105.0699; 91.0544; 60.4502 |
| **22** | Abscisic acid | 21.03 | $[M-H]^-$ | 263.1272 | $C_{15}H_{20}O_4$ | 247.9573; 241.1338; 216.7871; 208.3310; 203.1058; 184.7834; 163.0384; 141.9305; 125.4648; 72.7068; 56.3987 |
| **23** | Lancilimbnoid D | 22.33 | $[M-H]^-$ | 235.0607 | $C_{15}H_{24}O_2$ | 235.0980; 191.1070; 163.0754; 148.0518; 135.0439; 122.0366; 108.0204; 57.0333 |
| **24** | Curdione | 23.07 | $[M+H]^+$ | 237.1835 | $C_{15}H_{24}O_2$ | 237.1838; 159.1160; 135.1164; 121.1011; 107.0855; 95.0855; 79.0544; 67.0547; 55.0549 |
| **25** | Stachytriol | 23.83 | $[M-H]^-$ | 255.0659 | $C_{15}H_{28}O_3$ | 255.0660; 213.0550; 171.0442; 151.0026; 107.0125; 83.0125; 65.0019 |
| **26** | Teucdiol A | 23.90 | $[M+Na]^+$ | 251.1637 | $C_{15}H_{26}O_2$ | 251.1636; 233.1532; 205.1584; 175.1115; 147.1166; 119.0856; 105.0701 |
| **27** | Dihydroartemisinic acid | 24.15 | $[M+H]^+$ | 237.1833 | $C_{15}H_{24}O_2$ | 237.2189; 219.1735; 208.9817; 194.2572; 177.4289; |

| | | | | | | |
|---|---|---|---|---|---|---|
| | | | | | | 159.1163; 145.1010; 119.0850;105.0698; 91.0543; 67.0546 |
| **28** | (+)-Nootkatone | 24.17 | $[M+H]^+$ | 219.1731 | $C_{15}H_{22}O$ | 219.1735; 180.8745; 159.1164; 145.1010; 119.0852; 107.0854; 91.0544; 81.0702; 67.0547 |
| **29** | Aristolone | 26.64 | $[M+H]^+$ | 219.1727 | $C_{15}H_{22}O$ | 219.1727; 184.9780; 145.1005; 135.0798; 121.0645; 109.0646; 93.0700; 91.0702; 67.0547 |
| **30** | Germacrone | 26.93 | $[M+H]^+$ | 219.1730 | $C_{15}H_{22}O$ | 219.1741; 188.9343; 159.1160; 145.1006; 135.0799; 121.0644; 105.0697; 93.0699; 81.0701; 67.0546; 55.0548 |
